# An Exploration of Recent Intelligent Image Analysis Techniques for Visual Pavement Surface Condition Assessment

**DOI:** 10.3390/s22229019

**Published:** 2022-11-21

**Authors:** Waqar S. Qureshi, Syed Ibrahim Hassan, Susan McKeever, David Power, Brian Mulry, Kieran Feighan, Dympna O’Sullivan

**Affiliations:** 1 Department of Computer Science, Technological University Dublin, D07 EWV4 Dublin, Ireland; 2 Pavement Management Services Ltd., H65 PD37 Athenry, Ireland

**Keywords:** deep learning, image segmentation, pavement surface condition index

## Abstract

Road pavement condition assessment is essential for maintenance, asset management, and budgeting for pavement infrastructure. Countries allocate a substantial annual budget to maintain and improve local, regional, and national highways. Pavement condition is assessed by measuring several pavement characteristics such as roughness, surface skid resistance, pavement strength, deflection, and visual surface distresses. Visual inspection identifies and quantifies surface distresses, and the condition is assessed using standard rating scales. This paper critically analyzes the research trends in the academic literature, professional practices and current commercial solutions for surface condition ratings by civil authorities. We observe that various surface condition rating systems exist, and each uses its own defined subset of pavement characteristics to evaluate pavement conditions. It is noted that automated visual sensing systems using intelligent algorithms can help reduce the cost and time required for assessing the condition of pavement infrastructure, especially for local and regional road networks. However, environmental factors, pavement types, and image collection devices are significant in this domain and lead to challenging variations. Commercial solutions for automatic pavement assessment with certain limitations exist. The topic is also a focus of academic research. More recently, academic research has pivoted toward deep learning, given that image data is now available in some form. However, research to automate pavement distress assessment often focuses on the regional pavement condition assessment standard that a country or state follows. We observe that the criteria a region adopts to make the evaluation depends on factors such as pavement construction type, type of road network in the area, flow and traffic, environmental conditions, and region’s economic situation. We summarized a list of publicly available datasets for distress detection and pavement condition assessment. We listed approaches focusing on crack segmentation and methods concentrating on distress detection and identification using object detection and classification. We segregated the recent academic literature in terms of the camera’s view and the dataset used, the year and country in which the work was published, the F1 score, and the architecture type. It is observed that the literature tends to focus more on distress identification (“presence/absence” detection) but less on distress quantification, which is essential for developing approaches for automated pavement rating.

## 1. Introduction

**Figure 1:**
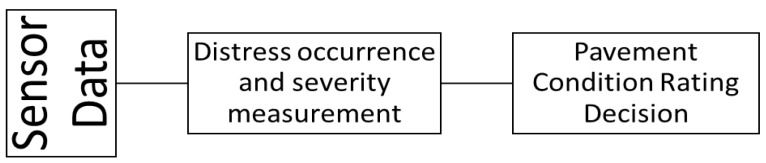
Pavement condition rating process.

Two vital elements of road pavement (referred to as pavements in the rest of this paper) management are inventory management and periodic condition evaluation; both are used to set future priorities for pavement construction management and maintenance. In this paper, pavement refers to hard surfaces used for motor vehicles. A complete pavement management system consists of inventory data collection (i.e., width, length, shoulder, and pavement type) and pavement characteristic assessment, i.e., (roughness (ride), surface condition (distresses), surface skid resistance, pavement strength, and deflection). The current pavement networks, including motorways across a country, are developed and modernized over centuries. The construction, width, and length of a pavement depend on the traffic it will carry and the type of connection it will make. They are classified into different categories; for example, in Ireland, they are classified as motorways, national primary, national secondary, regional roads, and local roads [1]. A common way to periodically evaluate surface condition, including distresses on a pavement network, is for the civil authority to conduct a visual surface condition assessment and a ride smoothness test. Surface condition is assessed through visual surveying and usually consists of three steps: (1) pavement condition data collection, (2) distress identification and quantification, and (3) assigning a pavement rating index to a stretch of a pavement using a standard rating scale (e.g., pavement surface evaluation rating-PASER [2]) that is typically localized to a specific geographical region [3]. Figure 1 gives a complete picture of the three-step process. The data collection is followed by distress occurrence, severity measurement, and pavement condition rating decisions.

Data collection, the first step of surface visual assessment, is usually carried out by specially adapted vehicles (or, more recently, on devices such as smartphones [4] or unmanned aerial vehicles) for visual surface surveying. The vehicle is fitted with a computer, Global Positioning System (GPS) sensor, and an imaging sensor. In step 2, pavement distresses are identified and quantified using their shape, size, and texture. Due to environmental and geographical conditions and the actual pavement construction process, pavement distresses may vary in shape, size, and texture. Variations can also be caused by different image capture technologies and the placement of sensors in specialized vehicles used to collect pavement data. In step 3, a rating is assigned to a stretch of pavement based on distress identification and quantification from step 2. A rating is applied to an initial stretch after inspection and then will be adjusted along the road if the pavement surface changes noticeably. The length of the stretch of road typically ranges from 50 m to 200 m, while the width of the stretch ranges from 4 m to the entire width of the road. The rating is performed directly by civil authority staff or subcontracted to private companies. Civil authorities use this condition rating to estimate pavement service life and treatment measures to improve the condition.

Maintenance and improvement of pavements are expensive. For example, Ireland’s government spent 850 Million Euros in 2021 to improve and maintain local, regional, and national primary and secondary roads [5]. There are 5413 km of national highways (primary, secondary, and motorways), 13,124 km of regional roads, and 81,300 km of local roads in Ireland. It totaled 99,830 Km of road network in 2018 in Ireland, meaning 95% of the road network in Ireland consists of regional and local roads [5]. Moreover, it takes most of the year to complete mechanical surveys on the national highways which are only 5% of the network, therefore we need a quicker method for the other 95%. Manual rating requires cognitive skills built through extensive training and experience. It is also impossible for a manual rater to transverse the whole road network across the country in a specific time. It is a challenging process and prone to errors. To make the process faster, more economical, and reliable, researchers have investigated automated processes for pavement condition evaluation, usually based on computer vision, machine learning, and, more recently, deep learning [6,7,8,9]. In recent years, researchers have reviewed different data acquisition technologies, including 1D-sensors, 2D-sensors, and 3D sensors, to automate pavement conditions [10,11,12,13,14,15]. Commercial solutions for automatic pavement assessment with certain limitations exist; the topic is also a focus of academic research. More recently, academic research has pivoted toward deep learning, given that image data is now available in some form. However, research to automate pavement distress assessment often focuses on the regional pavement condition assessment standards the country or state follows.

This paper contributes a list of significant pavement condition rating indices (segregated based on granularity and measurement criteria) used in various parts of the world. A comprehensive list of distress for asphalt and concrete roads is presented and segregated into six main groups. Commercial solutions for data capture and assisted image analysis are reported along with their limitations. We then present a comprehensive list of publically available datasets along with a link to download, which is segregated based on view type, type of distress, resolution, type of ground truth, number of images available, and country of origin. The review of recent (2018–2022) deep learning techniques for pavement distress detection, classification, segmentation, and direct pavement rating classification is presented. We segregated the literature in terms of the camera’s view and the dataset used, the year and country in which the work was published, the F1 score, and the architecture type that helps identify the latest trends. We observe that much of the literature focuses on automating step 2—distress identification and quantification- while there is less emphasis on automating step 3—automatically computing a pavement rating. We observe that the criteria a region adopt to make the evaluation qualitative depend on factors such as pavement construction type, type of road network in the area, flow and traffic, environmental conditions, and region’s economic situation.

This paper is organized as follows. Section 2 explains the type of pavement surfaces, the types of distresses, and pavement rating indicators used around the world, including their advantages and limitations. Section 3 reviews data collection techniques for visual pavement inspection and commercial practices. Section 4 generalizes an automated rating system and publically available dataset and reviews classical and deep machine learning approaches. Then, we discuss the limitations of an AI-based automated pavement rating system. Finally, we conclude in Section 5.

## 2. Pavement Surface Types and Distress Assessment Indicators

This section briefly explains various pavement types, visual pavement distresses, and pavement assessment indicators.

### 2.1. Pavement Surface and Distress

Pavement or road surfaces can be categorized into four general classes, i.e., asphalt, concrete, gravel, and brick and block [16]. Asphalt, also known as flexible pavement, is widely used to construct national, regional, or local roads across the road network and has different sub-categories depending on its construction. Over 90% of the total European road network has an asphalt surface. Concrete surfaces are usually used in urban environments and can be subdivided into joined cement concrete and continuously reinforced concrete surfaces [17]. Concrete pavements are expensive and time-consuming to construct, but they are typically more potent and durable than asphalt roadways. They are more common in the USA; for example, approximately 60 percent of the interstate system in the USA is concrete. Pavement condition assessment considers several pavement characteristics, i.e., roughness, surface condition (distress detection), surface skid resistance, and pavement strength. Surface condition plays a significant role in pavement assessment, which requires pavement distress detection and quantification. Pavement surface distresses that occur in different geographical regions can be divided into six groups, i.e., cracks, surface openings, surface deformation, surface defects, joint deficiencies, and miscellaneous distress [17,18] (see Table 1).

Most of these distresses can be detected generally through visual inspection (standard practice) of pavement surfaces, and their severity and quantity can be recorded using manual measurement tools [17]. Visual distresses appears on the surface due to wear and tear, which may indicate a fault in the construction. It may appear differently in rural and urban regions, depending on the surface type, the severity (low, medium, high) of the underlying problem, and other environmental conditions.

### 2.2. Pavement Assessment Indicators

Measuring different pavement characteristics is essential in long-term pavement performance incorporating all or a subset of pavement characteristics to conduct pavement assessments. These condition rating systems vary from country to country (or within a state in the USA), considering local variations, the characteristics of the pavements, and economic conditions.

Pavement characteristics that are generally separately measured include pavement roughness, a vital pavement characteristic measured on a rating index known as the International Roughness Index (IRI) [19]. It is estimated in a moving vehicle from a longitudinal pavement profile with sensors capable of measuring vertical movement [20,21]. Another essential characteristic is transverse deflection, also known as rut depth, measured manually or using sensors that generate transverse pavement profiles [9]. Visual pavement condition assessment requires distress detection and quantification to measure pavement conditions and is more reliable than other methods are. Engineers and professionals have proposed several standards for visual surface assessment, such as Pavement Surface Evaluation Rating (PASER) [2], Pavement Condition Index (PCI) [19,22], Pavement Surface Condition Index (PSCI) [23], and the Road Condition Indicator (RCI) [24]. Table 2 lists different pavement condition ratings used around the world. The standard ratings of various regions differ in scale granularity, formula to estimate a value on the rating scale, and data acquisition procedure.

The earliest work in creating a standardized condition assessment scale dates from the 1960s in the United States [25]. The scale used two pavement characteristics-pavement roughness and visual surface distress identification, to determine the Present Serviceability Index (PSI) ranges from zero (very poor) to five (very good condition). A roughness index was carried out by 3–5 individual raters trained to qualitatively estimate pavement roughness by driving a vehicle on the pavement. It was followed by visual inspection for cracks, patches, and potholes. These two were then combined mathematically to calculate the PSI score (0–5) [25].

Over the years, data acquisition techniques have evolved; different pavement condition assessment ratings have been proposed that mainly focus on assessing the different types of pavement characteristics, their quantity, and their effect on the overall condition of the pavement. PASER is a direct rating on a scale of 10–1 (9–10 is excellent condition, while 2–1 is extremely poor). On the other hand, the ASTM standard for pavement is PCI, a rating on a scale of 100–0 (85–100 is a good condition, while 0–10 is completely deteriorated. It is mathematically based on distress occurrence and severity level. The Irish PSCI [18,23,26] rating is on a scale from 1–10, similar to PASER, where index-1 is the lowest (surface completely worn out or failed), and index-10 (no distress, new pavement) is the highest. It covers flexible urban pavements, urban concrete pavements, and flexible rural pavement separately. PSCI ratings are given to continuous stretches of pavements with similar conditions, with 200 m being the minimum length to have their distinct rating [26]. In the United States, the Federal Land Transportation program recommends visual distress detection based on PASER for direct pavement condition evaluation [27]. Some transportation departments (or road authorities) that use scales similar to PCI use a subset of the visual distresses and roughness index to calculate the PCI rating. For example, the New Zealand Road Assessment and Maintenance Management System (RAMM) assigns a CI (Condition Index) from 0–100 (0–Excellent—100–Failed); it includes a visual inspection of not only the pavement but the surface water channels along the pavement [28]. China uses the Chinese Pavement Condition Index (CPCI), a scale similar to PCI, and considers cracking, raveling, potholes, rutting, and roughness. Japan used the Maintenance Control Index (MCI) until 2005, a function of cracking, rutting, and roughness, on a scale of 10 to 0 [29]. After 2005, the Ministry of Transportation Japan has used RRI, which is a function of cracking ratio, rutting depth, and International Roughtness Index [29]. A similar index is used in Tajikistan under Japan International Cooperation Agency [30].

The RCI is a rating from 1–4 (with 1 meaning no physical deterioration, while 4 is severe deterioration), adopted in England, Wales, Scotland, and Northern Ireland, and fuses visual condition and gauging parameters of pavement condition [24]. In Germany, the RMA (Road Monitoring and Assessment) protocol rate the pavement into four categories based on visual distresses [31]. Some states use four classes in the USA, i.e., Good, Fair, Poor, Very-Poor, as a condition scale based on the original PSI rating. In some countries, such as India and Brazil, a visible pavement distress condition rating on a scale of 0 to 3 is used [32]. Ratings are based on cracking, rutting, raveling, patching, and potholes, while roughness is not considered [3]. Pavement condition surveys of national and local roads are commonly conducted annually, every two years, or every five years in different regions across the world (for example, in Ireland, they are conducted every two years, while in Florida, state highway surveys are completed annually [33]). Therefore, these survey methods should be quick, fast, reliable, and economical.

In summary, different regions have different ways of performing pavement condition rating; some take roughness and visual condition combined to assign a rating from a standard scale (e.g., China, Japan, and some states in the USA), while others rate only a subset of visual distress (e.g., UK, Ireland, Brazil, Germany, New Zealand, India, and some states in the USA). Some of these indices are very granular (1 to 100) such as PCI in some parts of USA versus that (0 to 3 scale) used in Brazil/India. The choice of scale has evolved with economic prosperity and maturity of the road network.

## 3. Data Acquisition Process and Commercial Practices

This section describes how the data is acquired for pavement distress assessment and current commercial practices.

### 3.1. Data Acquisition Process

**Figure 2:**
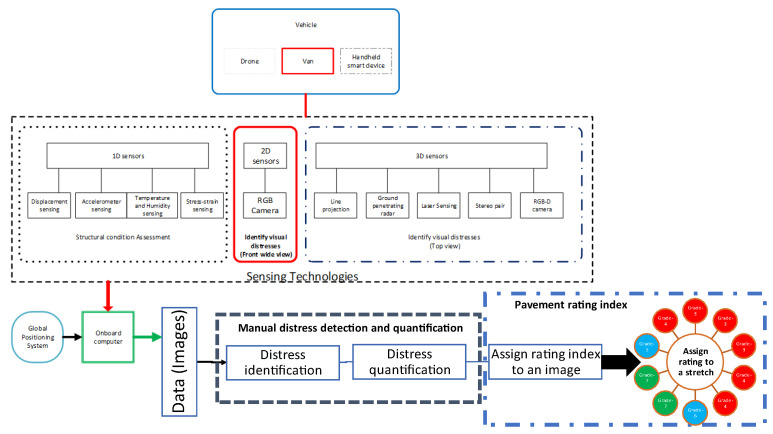
Different forms of data acquisition for pavement condition assessment that can be adopted.

Different sensing technologies, sensing positions, and vehicles have been used to capture data to assess pavement conditions. The choice of technology depends on economic factors, availability of resources, and pavement characteristics to be measured [4,34]; the sensor’s position depends on the sensing technology used to acquire data for pavement condition assessment [18,26]. Figure 2 lists three types of sensing technologies available for structural monitoring and distress detection that can be integrated into vehicles with a GPS.

To measure the pavement surface’s vibration, deflection, displacement, stress, temperature, or humidity, 1D or point sensors are usually used for structural condition assessment, which is an indirect method of pavement surface condition assessment. Data from 2D or 3D sensors is generally used for visual distresses identification and direct pavement condition assessment. 3D sensors, including laser imaging, stereo pair, and ground penetrating radar, are used obtained from the top view of pavement. In contrast, 2D sensors, including RGB (color) cameras, are used mainly in the frontal wide-view camera configuration.

Recently, research in [35,36,37,38,39,40,41,42] has shown promise in using aerial vehicles to help detect visual distress, such as potholes, cracks, and aging on asphalt pavement. Data collection using aerial imagery poses other difficulties, such as occlusion due to ongoing traffic, permission to fly in urban areas, and lower ground sampling distances. However, it does have limited use in pavement condition assessment, especially on airport runways [41]. Using a laser or color camera mounted at the back of the vehicles, a top view of the road, as used by [43,44,45,46,47], focuses on crack detections and potholes. A wide-angle view of the road, using a camera mounted on the front of the dashboard or top of the car, as used by [32,48,49], is used for detecting types of cracks, potholes, and types of surfaces and surface ratings.

Hand-held mobile cameras, as used by [47,50,51,52,53] have significant utilization in road surface distress detection. The top-view camera setup provides a better ground sampling distance than the wide-view setup, while the wide-view is much quicker as it covers more area per image. Thus, the literature review highlights that different camera capturing techniques for visual distress detection have been used: frontal wide-view, top-view, hand-held smartphone, and aerial view.

### 3.2. Current Commercial Practices

**Figure 3:**
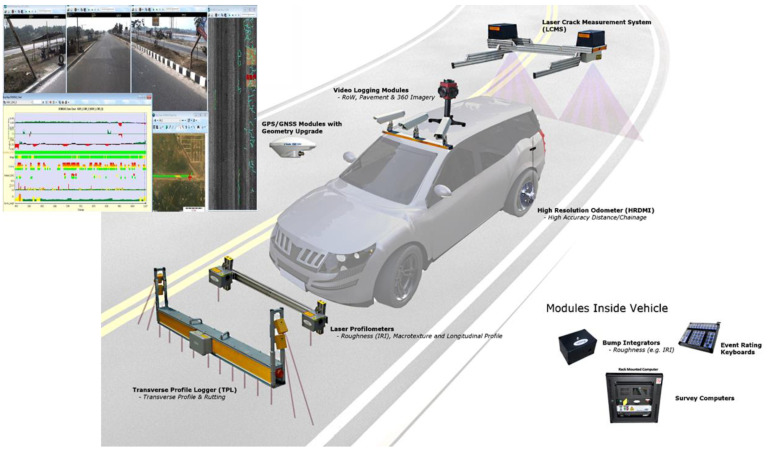
Picture of a particular commercial vehicle, typical sensors attached for capturing pavement images, and output shown by the software. This image is of a customizable vehicle reproduced from the website: https://romdas.com/romdas-dataview.html [54] (accessed on 15 November 2022).

Many commercial systems are available for image data collection for pavement condition assessment. These systems are reconfigurable and can be customized to carry different data sensors and inbuilt data analysis software for manual or semi-automated rating. The inbuilt software uses automated image analysis techniques to detect and quantify visual cracks for a pavement rating system. This section discusses currently available reconfigurable commercial systems used in different regions for data collection and assessment. A commercial vehicle usually consists of a GPS/GNSS module, transverse profile logger for rutting, laser profilometer for roughness estimation, high-resolution odometer, laser cracking measurement system, video logging modules for frontal wide-view capture, bump integrator, and an onboard computer for recording data (see Figure 3).

PaveVision3D [12] is a system that contains a data vehicle, an automated surface imaging system capable of conducting a complete lane width distress-detection survey at 1-mm resolution at a speed up to 100 KM/h. It uses a top-view approach with laser scanners and an intensity camera looking down on the pavement. It has dedicated software for crack identification, optional software and hardware for laser rut measurement, and laser roughness measurement. Pavemetrics [33] provides a similar solution called Laser Crack Measurement System (LCMS), which uses 3D laser scanners fitted on a vehicle. The LCMS software can geo-tag, measure, detect and quantify cracks, potholes, bleeding, shoving, raveling, and roughness. It can capture one lane of the pavement with a 1-mm resolution and a speed of 100 KM/h. The automated IRI and distress detection reports produced by LSTM comply with ASTM and the American Association of State Highway and Transportation Officials (AASHTO). These specialized vehicles are costly, and the distress detection software is calibrated for national highway pavement conditions in the USA or Canada.

In England, Wales, Scotland, and Northern Ireland, the pavement maintenance authorities use TRACS (Traffic-speed Condition Surveys) and SCANNER (Surface Condition Assessment for the National Network of Roads), which consists mainly of a laser scanner mounted on the front and back of a van giving a top view of the pavement surface. ROMDAS [54] is another customizable data-capturing solution that can provide both top-view using laser scanners for crack measurement or frontal view using the color camera for other detecting other distresses. STIER [55] is a customizable vehicle with a top-down stereo vision monochrome camera and a frontal view camera for data capture. It uses its software to detect distresses defined by German FGSV regulation, i.e., cracks, potholes, inlaid patches, applied patches, open joints, and bleeding. This information is then used to rate roads into four categories [31].

PMS video survey van [56] is equipped with distance-measuring sensors and a GPS sensor attached to the onboard computer to provide accurate distance measurements. A frontal wide-view video camera mounted on the dashboard of the pavement surface offers a high-quality compressed video stream using a state-of-the-art compression algorithm to retrain high definition (1920 × 1080) at minimum storage space. The real-time software integrated with the onboard computer provides options for the expert to rate the pavement condition on the go or record the video for offline manual PSCI rating. The ground sampling distance is lower than cameras providing top-view imaging. However, it can cover the whole pavement in one direction on multi-lane pavements with imaging every 5 m.

The speed of data collection through the PaveVision3D, Pavemetrics, and Ricoh (with a top-view 3D laser camera) [57] compared with less expensive dedicated vehicles [56] (with a high-resolution frontal wide-view camera) is comparable. However, the wide-view camera systems can cover more lanes than the top-view systems due to frontal coverage. The amount of data generated for records and further computation using top-view vehicles is more than from vehicles that use only frontal view for paving rating. The methods using 3D laser sensors (PaveVision3D, Pavemetrics, or RICOH) produce better ground sampling distance per pixel than frontal wide-view images. Still, the confidence in the final output of such reliable vehicles is much more than using a GoPro camera or a smartphone, as they do not have a customized processing unit to fuse readings from different sensors such as GPS and distance measuring sensors.

Therefore, a vehicle with a wide-view camera in front of a dashboard without external sensors (laser scanners or profilers), is more economical for an extensive network of local and regional roads with less maintenance requirement. It requires less storage to record pavement images by compromising spatial resolution; however, enough distress information to manually rate a pavement condition on a standard scale. On the other hand, vehicles with a top-view camera and external sensors are recommended for national highways and motorways [2]. They provide a higher spatial resolution, better for distinguishing different types of cracks and patches. Such vehicles have higher maintenance costs and would not be cost-effective when driven on the regional or local road network.

The commercial solutions discussed are usually limited to automated data capture and semi-automated analysis for distress identification and quantification, followed by an assisted or manual pavement condition rating assessment for a stretch of pavement. Some companies in the USA and Japan do provide automated solutions for pavement condition ratings. RoadBotics [58] working locally on USA roads, use a limited version of PASER [2], i.e., they rate pavement from 1 to 5, with 5 being the lowest rating. An automated rating system from Ricoh [57] estimated the amount and location of cracks on a 50 cm × 50 cm patch and has adopted its rating system for Japanese roads based on PCI. The automated solutions for frontal wide-view and top-view are still evolving toward robustness and generalization and require calibration and transfer learning with local data.

In summary, there is no off-the-shelf solution for automated pavement condition rating. Most of the existing commercial solutions usually provide automatic data and image-capturing solutions, while their ability to detect and quantify distress from images is limited to a few distresses. The limited automated solutions for intelligent distress detection (identification and quantification) from imagery require recalibration to capture regional variations in the pavement distresses for shape, size, or texture due to environmental conditions. These automated solutions also do not support adaptation to different pavement condition rating standards used by different regional and local authorities. The choice of imaging technology for visual inspection depends on the type of distress, environmental conditions, and economic factors and how adequate they are in identifying those distresses.

## 4. Literature Review on Automated Visual Pavement Condition Rating Systems

Automated visual pavement surface condition rating can be broken down into several processes: a pavement surface classification process, a distress detection and quantification process, prediction of a rating score computed using the type of distress detection and its quantification based on a standard rating scheme, and predicting the rating for a given stretch of pavement based on majority voting scheme. The manual PCI system works similarly, i.e., it identifies all the individual distresses and quantities, calculates ‘deduct’ values based on each distress type, severity, and amount, and then generates an overall rating by subtracting the sum of the weighted deduct values from a perfect score of 100.

To generalize the above statement, let $D_{0}$ to $D_{n}$ be the 0 to nth distinct types of distresses, $A_{m}$ be the area of the mth instance of the nth distress, and $w_{n}$ is the effect or weight on the rating score of the nth distinct distress, then we can define the rating score of a pavement condition in an image using Equation (1).

(1) $$Score_{\left(10-1\right)}=normalize_{1-10}(\sum_{o}^{m}\sum_{o}^{n}w_{n}A_{m}D_{n})$$

where $D_{n}$ is the distress, and n is the type of distress. $A_{m}$ is the area in m_th instance of $D_{n}$ and $w_{n}$ is the weight of each n_th distress to overall score.

For decades, extracting useful information from images has been a task of computer vision-based systems. Early researchers used image processing techniques (such as gradient or change in intensity detection, color or intensity thresholding, and morphological processing) to extract useful information from the pixels [59] directly. We first present a few prominent image analysis techniques and their limitations. Then we present a brief history of the evolution of machine learning techniques, benchmarking, and state-of-the-art models deep learning models for pavement condition assessment.

### 4.1. Evolution of Machine Learning in Computer Vision

With the development of machine learning algorithms such as K-mean classifiers [60], support vector machines (SVM) [61], ANN (artificial neural networks) [62], and many others [63], researchers started using hand-crafted features such as SIFT (scale-invariant feature transform) [64], ORB (Oriented FAST and rotated BRIEF) [65], or AKAZE [66] to uniquely describe an image, object, or region of interest. Image processing algorithms use these features to learn to classify, detect uniquely, or segment objects, areas of interest, or images. Hand-crafted features and classical machine learning provide robustness across scale, lighting, rotation, and other environmental conditions. Advancements in machine learning, including the development of dense neural networks [67], convolutional neural networks (CNN) [68], and more recently, Transformers [69], has provided solutions for computer vision tasks, which are more robust to changes in the input data and are coined as ‘deep learning’ computer vision or image analysis techniques. Handcrafted features are automatically extracted for a particular computer vision problem using deep learning algorithms.

### 4.2. Automated Distress Detection and Identification

The review of the literature tells us that researchers have investigated pavement distress detection using different imaging technologies, computing suitable features, and learning data models to detect, classify, or segment distresses over the last decade. In [7], the authors listed technologies to enable researchers to choose the imaging technique for pavement stress detection. They mentioned the state-of-the-art methods using image processing techniques for crack detection and potholes detection while highlighting the problems that need to be investigated, including pavement texture detection, temperature segregation detection, rutting detection, and joint faulting detection. The distress identification literature can be segregated into image processing, classical machine learning, and deep learning techniques.

#### 4.2.1. Publicly Available Datasets

Over the years, researchers have made available datasets for benchmarking automated distress detection systems, mainly covering different types of cracking and potholes [4]. Only a few focus on other distresses or visual pavement classification. In [14], the authors have reviewed different methods to detect pavement surfaces and highlighted different benchmarks for pavement surface detection. In [4], the authors list contributions to existing publicly available pavement image datasets for distress detection. These very limited datasets can be categorized based on the view angles (top-view, wide-view, hand-held), and imaging technologies (3D or intensity), mainly focused only on a subset of distress types (different crack types, potholes, and patches) found locally in the geographical regions (USA, China, India, Japan, Czech Republic, Brazil, Italy, and Mexico). In [70,71], the authors generated a pavement distress detection dataset using images available from Google APIs. The images available through Google APIs give both top-view and wide-view images; however, the images are old, captured over the years, and not labeled for pavement distress. The authors in [4] highlight that most of the literature on distress detection is based on image datasets not publicly available. Table 3 summarizes the current publically available datasets for distress detection and pavement condition assessments. The datasets are used as benchmarks to verify the crack segmentation algorithms include CrackTree200 [72], Crack500 [73], CrackForest [74], and Agile-RN [70]. Though several researchers have used it for verification of their deep learning-based architectures; however, they are limited in terms of covering various shapes, sizes, and textures of cracks formed due to different environmental conditions.

GAPS (German Asphalt Pavement Distress Dataset) used by [31,71] provides a top-view, good quality, close range, high-resolution dataset (approx ~2468 images) for surface distress identification which trained operators to label in the field. Six different distress defined by German FGSV regulation [71], i.e., cracks, potholes, inlaid patches, applied patches, open joints, and bleeding, are labeled in the images using a bounding box. The dataset is limited to only a few distresses regulated by German FGSV and does not contain severity levels of these distresses.

The second main contribution to the distress detection dataset is by [75], which has three different variants, namely, RDD2019, RDD2020 [75], and RDD2022 [76]. The dataset contains frontal-view images that are mainly labeled using a bounding box for four distresses, i.e., alligator, transverse and longitudinal cracks and potholes. The 2019 variant contains images of Japan, while the 2020 variant contains images from India and the Czech Republic. The 2022 variant contains images from China, Norway, and the USA. The dataset may be prone to labeling errors as it is labeled using crowdsourcing by labelers, not an expert in the field. A similar dataset for cracks with a wide-view camera located at the back of the vehicle is contributed by [77].

Two frontal view datasets focus on pavement rating; the first is the Paris-Saclay and the second is the Road Quality Dataset (RQ) [78]. The Paris-Saclay dataset [79] is annotated for pavement condition rating for a stretch of a road based on PASER for New York roads. The frontal-view images are extracted from Google Maps API, while the ground truth annotation for each stretch is extracted from the pavement condition rating of New York in [80]. The ground truth annotation contains the street index, the number of images in the street, the PASER rating for each street segment, and a rating of Good, Fair, and Poor for each street segment. A similar image dataset can be extracted from Google images for Oakland, USA, while the street segment pavement rating based on PCI can be generated from the database available at [81].

RQ Dataset [78] is a manually annotated frontal-view image for pavement condition index ratings based on six different condition ratings for the Czech Republic. The pavement condition rating criteria are defined in [78], while the images are obtained using Google Maps API. FHWA-LTPP [34] is another image-level classification resource composed of for five distress (alligator, longitudinal and transverse cracking, deflection, and longitudinal profiles) captured from different states of the USA.

#### 4.2.2. Image Processing Techniques

Techniques using decision-based rules and image processing mainly focus on crack segmentation and identification. The authors in [12] described different image processing techniques for edge detection to find surface defects and segregated the recent literature on pavement stress identification using machine learning models into classification, object detection, and pixel-level segmentation problems. The authors of [82] proposed a modified Otsu-Canny edge detection algorithm for pavement crack detection. They evaluated the technique on a publicly available dataset Crackforest [83]. Peng et al. [84] proposed a double thresholding segmentation technique. After applying an enhanced Otsu threshold segmentation algorithm to eliminate pavement symbols in a runway image, they applied an adaptive iterative threshold segmentation algorithm. Lastly, the shape of the crack is achieved through the morphological denoising technique. In [85], the authors propose a multiscale local optimal threshold segmentation for pavement crack segmentation and crack density distribution. The method achieves better results than the optical threshold and global thresholding techniques. Zhao et al. [86] proposed an improved pavement edge detection method for crack identification. In [87], authors have used image processing, including thresholding, filtering, and morphological processing, to identify fatigue cracks. CrackIT [88] uses image pre-processing techniques before applying machine learning models for crack detection.

Image processing techniques are mainly applied to pictures with a top view of the pavement. Moreover, the early literature focuses on identifying characteristics of cracks or potholes. The image processing techniques are less robust to changes in intensity, noise, environmental factors, and pavement construction variations.

#### 4.2.3. Classical Machine Learning Techniques

Machine learning approaches for distress identification can be classified as an image or object classification problems, object localization or detection problems, or pixel-segmentation problems. Many classical machine-learning approaches have been investigated for crack detection, including [89,90,91]. Daniel et al. [92] proposed a method to detect and classify cracks and potholes on asphalt pavements. They offered a two-step approach, pavement defect detection and classification, and defect severity detection and evaluation. The second stage is important for an automated pavement condition assessment and computed defect severity for each defect by calculating the area of the blobs. The method achieved 86% classification accuracy for cracks and potholes.

Raveling is a common visual distress in asphalt pavements, which occurs due to the loss of surface stones. It is recognized visually by observing the change in the macrotexture of the asphalt pavement along the stretch of the pavement. The severity of raveling increases with a higher chip loss from the pavement surface. In [93], authors evaluated different classical machine learning techniques such as AdaBoost with decision trees, support vector machine, and random forest to detect and classify different levels of raveling severity. For data collection for raveling, they used 3D images from PaveVision3D [12]. They observe that random forest is better than other techniques, with a recall ranging from 86.9% for level -1 severity to 75.6% for level-3 severityVery little work is reported on raveling detection and severity classification. In [94], the authors highlight the limitations in generalizing classical machine learning methods for crack detection. In [4], the authors have listed many classical machine-learning approaches for distress detection. These approaches mainly focus on detecting fatigue cracks, longitudinal and transverse cracks, potholes, rutting, and raveling. The image dataset is mainly captured through the top-view camera on a specialized vehicle, a hand-held camera view, or a UAV. Different hand-craft features have been extracted in these techniques. Models such as K-nearest neighbor, support vector machine, artificial neural network, and random forests are used to train a pixel-classifier (image segmentation) or an object detector. The precision ranged from 65.8% to 99% and recall from 79.4% to 98% [4].

However, these evaluation parameters are not generalizable as they depend highly on the image capture process. The datasets used mainly contain localized cracks or potholes, do not have different severity levels and are limited to a particular pavement type in a specific geographic region.

In summary, the public datasets available are limited to certain distress and mainly annotated by presence or absence of the distress. The image dataset annotated for pavement rating indices is also limited and does not cover the full range of standard visual rating scales, i.e., PASER [2] and PSCI [18]. The dataset does not cover distinct types of distress (mentioned in Table 1), or different shapes and textures, which vary due to different viewing angles, camera sensors, and geographical locations. Therefore, the evaluation matrix based on these benchmarks is less helpful in developing real-world automated pavement condition assessment systems.

#### 4.2.4. Deep Learning Techniques

We focused on literature from 2018 onwards for deep learning techniques. The techniques are mainly broken into segmentation, classification, and object detection algorithms. Deep learning techniques, mainly convolutional or filtering layers, require a large amounts of data. Deep learning techniques are now widely used for computer vision tasks, including semantic segmentation, image classification, object detection, and image generation [95]. Deep architectures have also been used to solve classification hyperspectral imagery for remote sensing [96,97].

Deep learning algorithms or architectures mainly consist of two parts the feature extraction phase and a classification, segmentation, or detection phase. In simple terms, for a deep learning-based classification, the CNN provides feature extraction layers, and the dense neural layer is added to estimate a class based on a feature extracted by the CNN. In deep learning-based segmentation, the CNN provides a feature extraction layer and is termed an encoder, while a set of de-convolutional layers are added to obtain pixel-level classification and termed a decoder layer. In deep learning-based object detection or localization, the CNN is used for feature extraction, followed by region proposal layers for object detection bounding box on the original image [68]. The interlinked deep learning layers are usually termed as ‘architectures’ or, when referred to alongside the weights and biases, as models.

Distress detection using a deep neural network can be separated into object detection, segmentation, and classification-based approaches [4,8]. One major bottleneck for developing a model using deep learning is a good set of balanced training data for different distresses in the images, instances, and quantity [98]. In [99], authors present the first CNN-based raveling detection by training macro texture features obtained from the 3D images from PaveVision3D [12]. They achieved the highest accuracy of 90.8% for different raveling detection and an 85% accuracy for severity classification.

##### Classification Approaches to Distress Detection

Classification-based distress detection focuses on whether an image or part of the image is classified as a particular type of distress. The authors in [100,101] proposed a flexible pavement distress classification convolutional neural network (CNN) framework to classify whether a patch is a crack or not. The images used are taken from a hand-held mobile phone camera. They evaluated the accuracy of their approach by comparing it with different classification approaches. Aparna et al. [102] assessed the feasibility of hand-held thermal imaging for pothole patch classification. Image data is acquired under various lighting conditions with offline data augmentation. A residual CNN model with pre-trained weights gave an accuracy of 99.7% for pothole patch image classification with an image size of 224 × 224 pixels. Yusof et al. [103] proposed a multi-label classifier for crack-type classification, i.e., transverse, alligator, and longitudinal. The images were taken from a hand-held Nikon digital camera with a dimension of 1024 × 768 pixels. The image was broken into a 32 x 32 patch image to classify different crack types. The data collection was carried out for Malaysian pavements.

An average accuracy of 98% was achieved to classify crack types with a precision of 97%. In [104], authors presented an algorithm for occurrence and severity classification in images captured from a top-view camera of urban flexible pavements in Spain. Their occurrence detection is based on patch classification using ResNet architecture, while the severity classifier is also a ResNet architecture. Each image is cropped to remove the background, broken down into three smaller blocks, resized to 224 × 224 pixels, and labeled for six classes, i.e., alligator cracks, longitudinal cracks, transverse cracks, pothole, raveling, and patches. To determine the severity of four distresses, mainly longitudinal cracks, transverse cracks, potholes, and patches, they labeled each distress with a bounding box in each image block. Although there were multiple distresses in each image block, the smaller block size minimized the likelihood of having different types of distress in each block. For the distress occurrence stage, the classifier’s average F1-Score was reported to be 0.9262 on validation data, while the average Intersection of Union (IoU) was 0.729.

Researchers [31,105,106,107,108,109] have also used a similar patch-based approach, i.e., dividing a higher resolution image into small image patches to detect localized distress, i.e., distinct types of cracks and potholes. In summary, most classification-based approaches focus on identifying types of distress in an image patch of higher-resolution images. Localized distresses are investigated, i.e., potholes and cracks. The images are taken from a top view or a hand-held camera view; the data set is localized to only specific to one region. The number of image patches is reasonable in number with a limited higher resolution image from where the patches have been extracted.

Many researchers have used patch or image classification techniques for multiple distress detection. Researchers in [43] and [44] used top-view color images for the experiment and then used ResNet-based architectures to develop a model for multiple distress classification. The ResNet model used in [43] has an F1-score of 0.92, whereas the model used in [44] has an F1-score of 0.90 on their test datasets. An image classification technique for multiple distress detection is mainly used for bleeding, raveling detection, and severity classification by [110] for pavements in Iran and [99] for pavements in the USA. In [46], the authors used detection and segmentation algorithms to classify four different types of cracks and then segment crack pixels. They [46] observe a better pixel segmentation F1-score on the CrackForest dataset than others using a multiple image-resolution training strategy.

Most researchers are focusing on multiple crack classification and having a better a F1-score using an image from a camera with an orthogonal view of the pavement and high ground sampling distances (i.e., pixel per inch). The evaluation of patch-based classification approaches for distress and its severity classification is limited in the literature. Patch-based classification and identification of distress instances are helpful for localized distresses; the technique is suitable for images that capture the top view of the road. It is computationally less expensive than pixel-level segmentation approaches. Table 4 provides a summary of the literature focusing on distress classification using either patch classification, image classification, or semantic segmentation.

##### Pixel Segmentation Approaches to Distress Detection

Segmentation-based approaches classify or label each pixel as a group or distress. Usually, distinct types of crack distresses are good candidates for pixel-level segmentation. The precise location of a crack can be determined using pixel-level labeling. In [111], the authors used U-Net architecture to segment crack pixels using a publicly available crack image database. The number of input training and test images is minimal; the experiment shows promise to segment crack pixels. In [112], the authors summarize a review of 68 manuscripts covering deep learning techniques for crack detection using segmentation. The authors evaluated eight segmentation models on 3D pavement images obtained from systems like [33]. They observed that FCN [113] and U-Net [114] performed better than others for 3D pavement images. In another attempt by [115], the author proposed a CNN-based segmentation algorithm named DeepCrack. The images are publicly available datasets of cracks from an intensity camera with a top view of the pavements with a dimension of 512 × 512 pixels. DeepCrack architecture, built with different scales and inspired by the SegNet network [115]. The authors in [100] used VGG-16 DCNN to detect cracks, by dividing high resolution images into smaller patches and use an image classification approach to detect cracks.

The authors have extensively evaluated DeepCrack with other state-of-the-art pixel segmentation models. The experimental result was an average F1-score of 0.85 for DeepCrack. The researchers in [116] have investigated the U-Net model architecture for crack segmentation; they used transfer learning techniques on pre-trained weights to train the classifier. The data on the concrete pavement is collected through a mobile phone at various locations at the Huazhong University of Science and Technology, China, with an image dimension of 512 × 512 pixels. The authors claim a higher accuracy and precision for crack pixel classification for concrete pavement types. In [117], researchers have proposed an asphalt pavement crack segmentation using a new CNN architecture. The data were collected from 12 cities in Liaoning province, China, through a hand-held mobile phone camera. The researchers have compared the results with existing segmentation models such as U-Net [118], SegNet [119], PSPNet [120], and DeepLabV3 [121]. The proposed model performance is better than the existing segmentation CNN architecture. Tang et al. [122] proposed an encoder-decoder network EDNet for crack segmentation. The network caters to quantity imbalance between crack and non-crack pixels. The images are taken from the top-view laser scanning camera to acquire 3D pavement images. The proposed method achieves an average F1 score of 97.80% and 97.82%.

Researchers focus more on cracks than other visual distress on the pavement surface when using deep learning techniques. One reason for this is that the crack is a fatigue on the surface that further disintegrates into potholes or total failure of the pavement surface. In Section 2.2, we observe that the occurrence and severity of visual distress, especially cracks, are essential to estimate the pavement conditions index. Table 5 summarizes crack segmentation and detection using deep learning. Researchers have mainly used encoder and decoder convolutional neural network architectures to segment crack pixels. Researchers in [94,107,109,123] proposed smaller customized CNN encoder-decoder networks, and the model is trained on smaller patches extracted from the higher-resolution image, while [115,124,125,126] used a modified UNET [113] based architecture, which is a fully convolutional network for semantic segmentation, and used a smaller resized image. Researchers combined three [127] and five publicly available datasets [51] for training their models and reported a lower F-1 score than previous ones using three deep learning architectures namely Holistically-Nested Edge Detection (HED), Richer Convolutional Features (RCF) and the Feature Pyramid and Hierarchical Boosting network (FPHB). For crack segmentation, the top orthogonal view is preferred over than front wide view of the pavement. The orthogonal view has the advantage of controlled lighting and higher pixels per inch; however, the disadvantage is of covering a lesser view of the pavement.

We observe that the biases and weights of the encoder (feature extraction part) are trained primarily from scratch instead of pre-trained on the ImageNet benchmark for developing a crack pixel classifier. Most crack detection and segmentation models are evaluated on publicly available datasets. Table 5 shows the test performance (F1- score) of different models developed using different architectures and training datasets. The deep-learning-based algorithms perform well when the test data is similar to the training images (i.e., from the same device); however, the performance degrades when the multiple training datasets are combined, or the test dataset is from a different capturing device and region. We also observe that automated segmentation deep learning algorithms using orthogonal images show a higher F1-score than the front or back view images. Methods like DeepCrack [115] holds promise to identify linear (transverse, longitudinal) cracks that are difficult to detect in patch-based methods.

##### Object Detection Approach to Distress Detection

Distress detection can also be approached using object detection. The approach is somewhat like patch-based image classification; however, the implementation is different in terms of the input and output of the CNN architecture. The object detection method can be used to find multiple object (distress) instances in a high-resolution image using CNN networks like Faster RCNN [135], the SSD MobileNet [136], or the YoloV3 [137]. Object detection-based techniques are usually used to detect different distresses, mainly including potholes, patches, cracks, and their various types and severities (see Table 6).

In [138], the author proposed a pothole detection system trained on images taken from a hand-held camera. The model was tested and compared with four object detectors. The authors observed that single-shot multi-box detectors (SSD) have higher accuracy but lower computational speed than YoloV3. YoloV3 fails in cases where the size of the pothole is small. In [139], researchers used Squeeznet architecture to train a model on image patches of size 64 × 64 extracted from two datasets with an orthogonal view of the pavement. The F1-score using the GAPs dataset was poorer than the F1-score obtained on the custom dataset obtained in the USA. Researchers in [45,140,141,142,143,144,145] used a version of the YOLO [137] architecture to train a model to detect different distresses. A crack severity detector for the top view of the pavement using YOLO with an average F1-score of 0.70 was proposed in [45].

**Table 6:** A list of publicly available datasets that used for distress detection analysis or pavement surface condition assessment.

S. No	Year	Country	Dataset	Architecture	Learning Method	Input Size	View	Channel	Distress	Size of Training Patches	F1-Score (or Accuracy *) of Test Data	Method -Type	Ref.
1	2018	Germany	GAPs/ ICIP	SqueezeNet	Scratch	64 × 64 64 × 64	top-view	Intensity	cracks, potholes	1,600,000/1,300,000	0.73/0.90	object detection	[139]
2	2018	China	private (China)	Faster RCNN	Transfer	-	frontal view	RGB	cracks, potholes	3200	0.88	object detection	[146]
3	2018	Timor Leste	private (Timor Leste)	Custom CNN	Scratch	200 × 200	frontal view	color	potholes detection	15,500	0.96	object detection	[147]
4	2019	China	private (China)	Faster RCNN	Scratch	1024 × 1024	top-view	RGB	crack pothole, bleeding, surface dots,	6498	0.89	object detection	[148]
5	2019	India	private (India)	ResNet50 + YOLO	Transfer	224 × 224	frontal view	RGB	pothole, pumps	5283	0.54	object detection	[140]
6	2020	China	LIST	YoloV3	Transfer	-	frontal view	RGB	crack, patch-crack, pothole, patch-pothole, net, patch-net, manhole	30,000	0.747 (excluding utility hole)	object detection	[141]
7	2020	USA	Paris-Saclay	YoloV2 for detection	Transfer	640 × 640	frontal view	RGB	longitudinal cracks, transverse cracks, alligator cracks, potholes, block cracks, reflective cracks,	5789	0.84	object detection	[142]
8	2020	South Africa	IBM-Hackathon	Custom 2-stage (LCNN object detection and PCNN for classification)	Transfer	352 × 224	frontal view	RGB	potholes	5000	0.936	object detection and classification	[143]
9	2020	China	private (China-Baidu)	Yolo3	Transfer	1024 × 512	frontal view	RGB	potholes, net-crack, cracks, patches	20,886	-	object detection	[144]
10	2020	USA	private (USA) - Google	Yolo2	Transfer	640 × 640	frontal view	RGB	reflective crack, transverse cracks, block crack, longitudinal crack, alligator crack, pothole	7237	0.84	object detection	[142]
11	2021	China	private (China)	Faster RCNN	Scratch	-	top-view	Laser 3D images	crack, pothole, patch	2208	0.95 * (MIOU)	object detection	[45]
12	2021	China	private (China)	YoloV5	Transfer	640 × 640	top-view	RGB	low-medium-high severity cracks	70,000	0.5	object detection	[45]
13	2021	India	private (India)	Custom CNN	Scratch	64 × 64	handheld	RGB	potholes detection	3424	0.97	object detection	[52]
14	2022	Lebanon	private (Lebanon)	YoloV3	Transfer	416 × 416	frontal view	RGB	pothole	344	0.6	object detection	[145]

Similarly, in [149], the authors experimented with thermal imagery and used object detection algorithms with an average precision of 91.15%. Maeda et al. [150] proposed an object detector based on SSD MobileNet and Inception V2 architectures. They achieved an average recall of 77% with a precision of 71% for potholes, alligator cracking, and blurry line marks. However, the ‘presence/absence’ detection is not very helpful for quantifying distress, which is essential for pavement surface evaluation.

Table 6 shows a summary of object-detection-based distress detection. The F1-scores indicate that the performance of the object detector deteriorates for multiple distress detection compared to detectors that detect one or two distresses. We observe that Yolo architectures promise to detect distresses from a frontal view of the camera; however, developing a robust model for a region will require calibration from the local distresses. Top-view, hand-held cameras, and wide-view images have been used in experiments. The object detection-based algorithm can be used for localized distress detection, such as alligator cracks and potholes. The localization and detection accuracy is better than the patch-based method. Recall or accuracy for detecting cracks (linear or edge) using a frontal view image is less when object detection networks such as Yolo [137] are used compared to top-view images.

#### 4.2.5. Automated Direct Pavement Condition Rating 

It is highlighted in the introduction of this paper that the primary purpose of distress detection and identification is to evaluate the condition of the pavement using a standardized scale. Distresses must first be identified to compute a rating for an extensive pavement network. Then the number of distinct distresses and their severity must be considered over a given stretch of pavement. Most research focuses on distress identification but falls short of computing a direct pavement rating for a stretch of pavement. One approach to computing direct ratings is described in [151], where the authors present a hybrid model of an object detector and semantic segmentation for classifying and quantifying distress severity on pavements and predicted PASER indices for each patch. The images are collected from Google Street View maps - 70-degree wide-angle views, and 90-degree birds-eye view images. Wide-view photos are used for crack and pothole detection, and top-view images are used to quantify crack severity. The results from the hybrid model are then fed to a linear and weighted regressor for predicting PASER indices to pavement patches. They trained YOLO to detect distress and used U-Net (based on a fully convolutional layer) to classify crack severity. The results from the two models are then combined to find the crack density per pavement defect. The results are then fed to a linear and a weightage regressor to label each image a PASER index. The photos are from USA pavements, and the PASER calibration set is minimal. The predicted PASER model fits with an R^2^ of 0.9382 or test data with a root mean square error of 10.45. One of the limitations of this research is the use of Google API images that are usually older. In this system, only two distresses are taken for the rating (cracks and potholes); however, in most practical scenarios, cracks, potholes, patches, raveling, and bleeding also need to be considered, requiring transfer learning for adding localized distresses further modification in the algorithm for raveling and bleeding.

In [152], the authors have presented an image classification approach to surface rating using a three-rating index-good, regular, and bad. The dataset used for the experiments is RTK [32], caRINE [153], and KITTI [154]. It classifies roads into three different types and three different ratings. The images are cropped to extract the region of interest that contains the road. Data augmentation is performed to increase the robustness and avoid overfitting. The authors used three convolutional layers, a flattening layer, and two fully connected dense layers to classify the road types into asphalts, paved, and unpaved. The classified images are then further passed through another classifier to estimate the quality of each road, as good, regular, and bad for each class. The surface type accuracy is reported as 98% for three types. The classification accuracy for the three quality types is 98% for good asphalt and 96% for bad asphalt. The precision of classifying the good class is 86.7%, while classifying the bad asphalt class is 81%. The number of rating indices is limited to three—good, bad, and regular, and they only relate to Brazil’s actual standard rating system. However, judging on a scale of 3 levels is not very useful in real life, where maintenance decisions are based on the overall rating, and individual distresses that lead to that rating. Moreover, it also requires further experiments to increase the number of image classes to be adopted for visual standards such as PASER or PSCI. The higher statistics of recall and precision are much easier to obtain if the images are simple-complex images with multiple distress and different quantities are much more difficult.

In [155] the author presented the complexity of manual PCSI practices. The author used pixel segmentation using a semantic segmentation CNN-based model from [156] to extract roads, marks, and background pixels. They analyzed state-of-the-art EfficientNet V2 [157] image classification approach for automating PSCI ratings. Each image in the training and test set has a ‘segmented’ pavement image, an ‘augmented’ image, and an ‘original’ image. Image height is cropped 250 pixels from the top and 50 pixels from the bottom to remove the sky and pavement pixels further away from the camera and pavement pixels too close to the camera. ‘Augmented’ image is computed by combining the pavement segmented intensity image, the pavement plus mark pixel intensity image, and the original intensity image. They used a combination of these images to evaluate the performance of the classifier. For a 10-class classification, the best model achieved an F1-score of 0.57, while a 0.73 for a five-class classification after combining adjacent classes.

### 4.3. Benchmarking and State-of-the-Art Models

During the last decade, researchers have developed benchmark datasets to evaluate deep learning models, especially CNN feature extraction layers [158], and images labeled for a particular computer vision task. The algorithm is known as a state-of-the-art model if the model’s performance matrix is the best if evaluated against benchmarks [159]. The website [160] gives a structured approach to finding state-of-the-art models for different computer vision, natural language processing, and signal processing tasks on the respective datasets. Improving the state-of-the-art models using benchmark datasets is one approach; however, recently, researchers have argued that an application-centric process must be followed for a deep learning solution. In [160], Hooker argues that chasing benchmarks is incorrect for evolving a machine learning model. Instead, smartly chosen training images specific to a particular application helps in better understanding for developing a deep learning-based solution as suggested by [161]. Across different subfields of AI, specifically in machine learning, current benchmarking practices tend to distort the development of fair and flexible AI systems for real-world scenarios. In [162], the authors systematically explored the limitations of influential dataset-based benchmarks, revealed the construct validity issue, pointed out the risk associated with their framing, and proposed alternative performance evaluation methods. The authors [162] have logically argued that the state-of-the-art performance of AI models on these benchmarks does not validate the general-purpose capabilities of models, particularly in visual and language understanding domains.

Therefore, benchmarking is a conservative approach to assessing general model capabilities due to limited task design, de-contextualized data, hidden biases, false performance reporting, and inappropriate community use in the machine learning context. These benchmarks are arbitrarily selected subsets of objects from the real world and cannot cover the domain knowledge for a particular application. It is recommended that along with recalibration or transfer learning with a localized dataset, alternative methods such as unit testing and failure mode analysis could measure the broader capabilities of an automated pavement rating system.

### 4.4. Limitations of AI-Based Automated Pavement Rating Systems

A country or region’s adaptation to a standard, or defining local variants for pavement condition assessment depends on environmental factors, local pavement distresses, and economic factors in the data collection process. The evolution in imaging technology, computational power, and CNNs have made pavement condition assessment through visual distress detection fast, quick, easy, and cost-effective for a country’s comprehensive pavement survey. In [9], the authors summarized different imaging technologies, types and sub-types of distress, and distinct levels of distress severity (i.e., low, medium, and high). The variation in shape, size, and texture is due to different severity levels of these distresses due to various weather conditions in different geographical locations [17]. The variation in data in different regions is not only because of changes in shape, size, and texture of distresses but also due to different imaging technology and placement of sensors in specialized vehicles. Automated condition rating for a pavement stretch depends on types and sub-types (severity-level) of distress and the amount of distress present in a particular stretch. The CNN-based automated decision tools depend on learning from statistical information present in images; therefore, data injected for learning needs to be centric to the problem domain, smartly sized, and less noisy [161]. The accuracies and precisions mentioned in the literature are reported on limited data sets, certainly not with complex images with multiple distresses of different shapes, sizes, or textures. Any automated rating system using imaging technology needs to be recalibrated (for example, using transfer learning techniques) for the regional distress to capture variation in shape size, the texture of distresses, and variation in light intensity. The highlighted environmental factors (such as rain, standing water, poor lighting, and moisture) play a crucial role in distress shape, size, and texture. Moreover, while imaging these distresses for pavement condition assessment, the algorithm is not generalizable for different geographical locations due to the distress’s environmental factors, shape, size, and texture.

Orthogonal views capturing the pavement requires expensive external 2D and 3D sensors mounted outside the back of the vehicle, which makes it expensive to maintain. It increases the budget for pavement condition assessment for a road network across the country, especially the local network. However, it captures images with controlled lighting conditions, which help in the automated detection and segmentation of cracks and patches. It is recommended for use on national highways and motorways. The frontal view capturing of the pavement requires low-cost cameras that can be mounted inside the vehicle, which makes it less expensive and lower budget. It captures a wide view of the pavement, which helps in the automated detection and classification of different distress types, including raveling, bleeding, different types of patches, cracks, and potholes. It is recommended to cover a bigger network of pavement surfaces, including local and regional roads. Another challenge for such approaches is the unavailability of very large datasets of labeled data—labeled images identifying multiple distress types and their severity levels are expensive to create, requiring both time and expert knowledge.

## 5. Conclusions

Technology and intelligent algorithms for automated pavement surface condition evaluation have evolved during the last decade. The literature indicates the experimentation in evaluating different imaging technologies (such as intensity, color, and 3D laser camera), imaging road views (top-view, wide-view, or hand-held), and developing a robust algorithm for detecting distinct instances of distresses in an image—moreover, very little work is found on pavement condition assessment rating. The current limitations include a lack of a general evaluation matrix to evaluate the robustness of the detecting algorithms for different shapes, sizes, and textures of distinct distresses in different geographical locations. The lack of algorithms for quantifying these distresses in images and, finally, for rating a stretch of pavement using a sequence of images to develop a real-world automated pavement condition assessment rating. In practice, a rating is assigned to a stretch (200 m or 100 m) of pavement instead of one image; different regions follow different assessment standards.

We found little work on automatically computing direct pavement ratings. The recent literature reviews pavement condition evaluation summarize imaging technologies and different machine learning approaches for distress detection and identification; however, they have limited insight into the correlation between standard condition rating practice to distress detection and its quantification. Road or pavement rating conditions depend on the type of distress and quantification, which changes (shape, size, and texture) with several factors, including environmental conditions (weather) and the pavement construction process. The highlighted environmental factors (such as rain, standing water, poor lighting, and moisture) play a crucial role in these distresses’ shape, size, and texture.

For data collection, the top view of the pavement and a wide-angle view of the pavement has been used for distress detection, identification, segmentation, and pavement condition ratings. Choosing an imaging technology for visual inspection depends not only on the type of distress but also on environmental conditions and economic factors and how adequate they are in identifying those distress. The top view gives a higher ground sampling distance but covers less area per image than wide-view images. Vehicles with external laser scanners and stereo pairs are more expensive to operate and maintain than vehicles with an internal high-resolution camera with a frontal view. In summary, there is no off-the-shelf solution for automated pavement condition rating. Most of the existing commercial solutions usually provide automatic data and image-capturing solutions, while their ability to detect and quantify distress from images is limited to a few distresses.

Many of the datasets available as benchmarks are limited only to cracks and potholes and are localized to a geographical location. Research on automated pavement distress assessment is often limited to the regional pavement condition assessment standard that a country or state follows. The criteria a region adopts to make the evaluation qualitative depends on factors such as pavement construction type, type of road network in the area, flow and traffic, environmental condition, and region’s economic situation.

Most of the automated image-analysis-based pavement condition assessment focuses on two primary distress, i.e., distinct types of cracks and potholes. Very few experiments can be seen in the literature on raveling or bleeding (see ([163,164]), which are forms of surface defects and contribute toward a unified pavement surface rating. Other surface distress, such as patching, utility patches, and utility cover, is seldom considered (see [165]). PASER (used in the USA and other regions) and PSCI (used in Ireland), the ratings 10-7, are decided based on the amount of raveling and bleeding alone. Similarly, the study of direct pavement ratings from images as a classification problem is limited, apart from [152] and [151]. The ‘presence/absence’ detection is not very helpful for quantifying distress, essential for pavement surface evaluation. Higher levels of recall and precision are much easier to obtain if the images are simple; complex images with multiple distresses, and their quantities are much more difficult. Automated distress detection and condition rating is not a time-critical process, it can be conducted offline, so accuracy and precision are more important than computational time.

In the future, automatically computing a rating for a stretch of pavement will need to combine several methods. For example, image processing techniques such as cropping may be required to remove objects such as the sky, buildings, cars, and sidewalks to prepare images for use by machine or deep learning models. Then, segmentation may be used to segment the distinct distress and use the number of pixels of each different instance to calculate the area of the distress. A similar approach could be implemented using object detection-based approaches to detect individual distresses. Distresses such as rutting and sag may require multiple images or a fusion of point sensor information to establish the presence of such stresses. Deep learning models will need to be calibrated (trained) to capture the severity levels of each distress for a local region where it needs to be deployed. The number of distresses and their severity can be used to compute a rating score averaged over a set of images for a given stretch of road. Advances in deep learning may allow computing a rating directly using image classification. Still, a lack of benchmark datasets containing various distresses for learning may hinder such approaches. Developing a benchmark dataset for a diverse set of distinct distresses and their severity levels is challenging, as it requires extensive data collection to capture different environmental conditions and regional variations. We propose that any automated rating system for pavement conditions using imaging technologies will require re-calibration (i.e., transfer learning) for the regional distress to capture variations in shape, size, the texture of distresses, and variation in light intensity.

## Figures and Tables

**Table 1:** A comprehensive list of distresses in asphalt rural flexible, asphalt, urban flexible, joined Portland concrete, continuously concrete reinforced roads, and segregation in six main groups and their sub types [16,17].

Surface Distress Group	Asphalt Rural Flexible	Asphalt Urban Flexible	Joined Portland Concrete	Continuously Concrete Reinforced
Cracks	Alligator cracking	Fatigue cracking	Durability cracking	Durability Cracking
		Block cracking		
	Edge cracking	Edge cracking	Corner breakups	Corner breakups and shattered slabs
	Reflection cracking at joints	Reflection cracking at joints		
	Longitudinal cracking	Longitudinal cracking (wheel path and non-wheel path)	Longitudinal cracking	Longitudinal cracking
	Transverse cracking	Transverse cracking	Transverse cracking	Transverse cracking
	Meander and slippage	Meander and slippage		
Surface Openings	Patches	Patches and utility patches	Patches and utility patches	Patches and utility patches
	Potholes	Potholes	Blow-ups	Blow-ups
	Surface disintegration	Utility hole defects	Utility hole defects	Utility hole defects
Surface Deformation	Rutting	Rutting	--	--
	Depression and bumps	Shoving, depressions, bumps, sags, and heave		
Surface Defects	Raveling	Raveling	Wearing	Wearing
	Bleeding	Bleeding	Polish aggregate	Polish aggregate
Miscellaneous Distresses	Lane-to-shoulder drop off	Lane-to-shoulder drop off	Lane-to-shoulder drop-off and separation	Lane-to-shoulder drop-off and separation
	Water bleeding and pumping	Water bleeding and pumping	Water bleeding and pumping	Water bleeding and pumping
Joint Deficiencies	---	---	Joint seal damage (longitudinal and transverse)	Joint seal damage (longitudinal and transverse)
			Spalling of longitudinal and transverse joints	Spalling of longitudinal and transverse joints

**Table 2:** A summary of different pavement condition rating systems used by regional road transportation departments or proposed by academics.

Type of Indicators	Granularity	Measurement Criteria	Standard Developing Body
Present Serviceability Index (PSI)	5 (Excellent)—0 (Essentially impassable)	A mathematical formula based on the severity of surface roughness, cracking, deflection	Illinois, Minnesota, and Indiana—AASHO Road Test (1961)
	Integer value		
Pavement Condition Index (PCI)	100–85 (Good)—0–10 (Failed)	A mathematical formula based on the occurrence, and severity of distresses, mainly crack and IRI	ASTM D6433—11
Pavement Condition Rating (PCR)			Alabama Department of Transport
Pavement Structural Condition (PSC)			Washington Department of Transport
Surface Condition Rating (SCR)			Georgia Department of Transport
Pavement Surface Evaluation and Rating (PASER)	10 (Excellent)—1 (failed)	A direct rating based on visual distresses	Wisconsin Transportation Information Center, University of Wisconsin Madison, USA
	Integer value		
Pavement Surface Condition Index (PSCI)	10 (Perfect)—1 (No surface)	A direct rating based on visual distresses	Road Management Office, Ireland
	Integer value		
Unified Pavement Distress Index for Managing Flexible Pavements (UPDI)	0 (Failed)—1 (Perfect)	A mathematical formula based on six visual distress	Civil Engineering Department, Clemson University, USA
Pavement Distress Index (PDI)	Good/Fair/Poor	IRI, rutting, cracking, and faulting are used to estimate PDI	Arizona Department of Transport
Pavement Performance Levels	Good/Fair/Poor	IRI, rutting, cracking, and faulting are used to estimate PDI	Kansas Department of Transport
Pavement Quality Index (PQI)	0 (Fail)—4.0 (Good)	A square root of the product of roughness quality index (RQI) and visual surface rating (SR)	Government Accounting Standards Board, Standard 34 (GASB 34). Minnesota
Condition Rating Score (CR)	1–59 (Very poor)—90–100 (Very good)	Mathematical combination of distress and ride quality (roughness)	Texas Department of Transport
Pavement Condition Index -2	1–100 (same as PCI)	A mathematical formula based on cracking index, riding index, and rutting/faulting index	IOWA STATE University Institute for Transportation
Pavement Condition	Good/Fair/Poor/Very/Poor	A pavement condition based on the international roughness index	New Hampshire Department of Transportation
Remaining Service Life (RSL)	Good/Fair/Poor	A superset rating is calculated based on PCI rating (0–100)	Colorado Department of Transportation
Chinese Pavement Condition Index	100–85 (Good)—0–10 (Failed)	A mathematical formula based on the occurrence, and severity of distresses, mainly crack and IRI	China
Maintenance Control Index (MCI)	10 (Good)—0–1 (Failed)	A mathematical formula based on cracking Ratio, Rutting Depth, and roughness	Japan (Until 2005)
Repair Requirement Index (RRI)	0-5 New – More than 12 (Lifetime over)	A mathematical formula based on International Roughness Index, crack rate coefficient, and pothole rank coefficient	Japan (after 2005) Tajikistan
Road Condition Index	1 (poor)—4 (Good)	A mathematical formula based on the occurrence and severity of visual distresses and roughness index	UK
Pavement Distress Condition Rating	Good/Fair/Poor	A rating is based on maintenance strategy and is a function of cracks, patches, and potholes	India
Condition Index (CI)	0 (Excellent)—100 (Failed)	A mathematical formula based on visually measured condition defects	New Zealand
RMA	1 (Poor)—4 (Good)	A mathematical formula based on the occurrence and severity of visual distresses and roughness index	Germany

**Table 3:** A list of publicly available datasets that can be used for distress detection analysis or pavement surface condition assessment.

S.No	Name	Distress	Ground Truth	Device	No. of Images	Resolution	Ch	View	Country	Link
1	Crack Forest Dataset (CFD)	crack	pixel-level	hand-held static	329	480 × 320	3	Top	China	https://bit.ly/3PMFWhl accessed on 20 October 2022
2	Amhaz Crack Dataset (Aigle_RN + ESAR + LCMS + LRIS = TEMPEST2)	cracks	pixel-level	vehicle with 5 sensors	66 (38 + 15 + 5 + 3 + 5)	991 × 462 + 311 × 462 + 768 × 512 + 700 × 1000 + 3249 × 1576 + 1127 × 1598	1	Top	France	https://bit.ly/3TdmOfB
3	CRACK500 and CRACK-500-B	cracks	pixel-level	hand-held static	500 + 1896	2560 × 1440 640 × 360	3	Top	China	https://bit.ly/3QPeAsx
4	GAPs-10m	22 classes	pixel-level	JAI Pulnix TM2030 monochrome cameras (vehicle)	20	5030 × 11,505	1	Top	Germany	https://bit.ly/3cnqI4X
5	GAPs	crack, pothole, inlaid patch, applied patch, open joint, bleeding	bounding box	JAI Pulnix TM2030 monochrome cameras (vehicle)	2468	1920 × 1080	1	Top	Germany	https://bit.ly/3cnqI4X
6	Paris-Saclay	pavement rating (1–3)	image	Google Streetview	700,000	640 × 640	3	Frontal	New York, USA	https://bit.ly/3pNlYc4
7	RDD2019	pothole, longitudinal crack, transverse crack, alligator crack, line markings	bounding box	mobile device moving vehicle	10,561	600 × 600	3	Frontal	Japan	https://bit.ly/3cqCKun
8	RDD2020 (excluding Japan)	pothole, longitudinal crack, transverse crack, alligator crack	bounding box	mobile device moving vehicle	11,000	720 × 720 (India) 600 × 600 (Czech)	3	Frontal	India Czech Republic	
9	RDD2022 (excluding RDD2020)	pothole, longitudinal crack, transverse crack, alligator crack	bounding box	mobile device moving vehicle	17,500	3650 × 2044 (Norway) 640 × 640 (USA) 512 × 512 (China)	3	frontal/top	Norway USA China	
10	DatasetCrackDeepa2022	cracks	pixel-level	hand-held static	3000	800 × 600	1	Top		https://bit.ly/3coASSY
11	RQ Dataset	pavement rating (1–6)	image	Google Streetview	7247	640 × 480	3	frontal	Czech	https://bit.ly/3pMYofi
12	CrackIT	crack	pixel-level	hand-held static	56	1536 × 2048	3	Top	Portugal	https://bit.ly/3RajLCR
13	EdmCrack600	crack	pixel-level	camera mounted on vehicle	600	1920 × 1080	3	Back	Canada	https://bit.ly/3ThzDW8
14	FHWA-LTPP	aligator, transverse crack, longitudinal cracks, deflection, IRI	image	camera mounted on vehicle (top and frontal)	-	2048 × 3072	3	frontal and top	USA Canada	https://bit.ly/3CzyNOO
15	bim-hackathon	potholes	bounding box	mobile camera mounted on vehicle	5676	3680 × 2760	3	frontal	South Africa	https://bit.ly/3RNGSDV
16	LIST	crack, patch-crack, pothole, patch-pothole, net, patch-net, manhole	-	camera on a moving vehicle	30,000	-	3	frontal	China	https://bit.ly/3qchLPd
17	CrackTree200	cracks	images	hand-held static	260	512 × 512	1	Top	China	https://bit.ly/3ARIEg6
18	CRKWH100	crack	images	hand-held static	100	512 × 512	1	Top	China	https://bit.ly/3QcPdzL
19	CrackLS315	crack	images	hand-held static	315	512 × 513	1	Top	China	https://bit.ly/3QcPdzL
20	APR	cracks	pixel-level	camera on a moving robot	19 + 14	1200 × 900 + 2040 × 2048	2	Top	China	https://bit.ly/3RwZF6y

**Table 4:** A summary of the literature focusing on distress classification using either patch classification, image classification, or semantic segmentation.

S.No	Year	Country	Dataset	Architecture	Learning Method	Input Size	View	Channel	Distress	Size of Training Patches	F1-SCORE (or Accuracy *) of Test Data	Method-Type	Ref.
1	2019	Itlay	private (Italy)	ResNet101	Transfer	224 × 224	Top	RGB	9 distresses (e.g., longitudinal cracks, transverse cracks, alligator cracks, potholes, patches,	12,728	0.92	patch-based classification for sliding window	[43]
2	2019	Germany	GAPs (Germany)	RestNet34	Transfer	160 × 160	Top	Intensity	cracks applied patches, inlaid patches, open joints, potholes	50,000	0.9041	patch-based classification for sliding window	[44]
3	2020	China	private (China)	customized (RCNN + FCN)	Scratch	75 × 75	Top	Laser 3D images	cracks, pothole, patch	2208	0.87	semantic segmentation	[45]
4	2020	China	private (China) + CFD	YoloV3 + UNET with ResNet34	Transfer	128 × 128 + 256 × 256 + 320 × 320	Top	RGB	longitudinal and transverse cracks, block crack, alligator and linear crack	16,780	0.906 (detection) 0.957 (segmentation)	instance detection and segmentation	[46]
5	2020	Canada	private (Canada)	customized U-Net	Scratch	1024 × 1024	Top	RGB	transverse and longitudinal cracks, alligator cracks, and block cracks	3000	0.984	semantic segmentation	[47]
6	2021	Iran	private (Iran)	SqueezeNet	Transfer	224 × 224	Frontal	RGB	bleeding detection and severity classification	800	0.98	image classification	[110]
7	2021	USA	private (USA)	ResNet18	Transfer	520 × 417	Top	Laser 3D images	raveling detection and classification	2500	0.915	image classification	[99]

**Table 5:** A summary of the literature reviewed that focuses only on crack segmentation and detection using deep learning techniques (2018–2022).

S.No	Year	Country	Dataset	Architecture	Learning Method	Input Size	View	Channel	Size of Input Patch	F1-Score (or Accuracy *)	Method	Ref.
1	2018	USA	LTPP-FHWA	VGG16	Transfer	2072 × 2048	Top	Intensity	760	0.9	image classification	[100]
2	2018	Vietnam	custom (Similar to CrackIT)	custom CNN	Scratch	100 × 100	Top	RGB	12,500	0.91	patch-based classification for sliding window	[109]
3	2018	China	private (China)	custom CNN	Scratch	256 × 256	Top	Laser 3D images	4000	0.98 *	patch-based classification for sliding window	[107]
4	2018	Vietnam	private (Vietnam)	custom CNN	Scratch	150 × 150	Top	Intensity	400	0.907	patch-based classification for sliding window	[123]
5	2018	France	Amhaz Crack dataset + CFD	custom CNN	Scratch	27 × 27	Top	RGB	898,764	0.8954 + 0.9244	patch-based classification for sliding window	[94]
6	2019	USA	private (USA)	custom CNN (encoder + decoder)	Scratch	1024 × 512	Top	Laser 3D images	3800	0.94	semantic segmentation	[128]
7	2019	USA	crackTree CRKWH100 CrackLS315	custom UNET (DeepCrack)	Transfer	512 × 512	Top	RGB + Laser	260	0.95 + 0.84 + 0.85	semantic segmentation	[115]
8	2019	China	CrackForest	U-Net with patch training	Scratch	48 × 48	Top	Intensity	20,000	0.874	semantic segmentation	[111]
9	2019	China	CrackForest + Aigle_RN	U-Net with residual block, attention Unit, and patch training	Scratch	48 × 49	Top	Intensity	142,000	0.92	semantic segmentation	[129]
10	2019	Korea	private (Korea)	custom CNN (ResNet +decoder)	Transfer	1920 × 1080	Front	RGB	427	0.74	semantic segmentation	[124]
11	2019	China	Aigle_RN + crackForest + APR	multi-scale fusion (unsupervised) learning	Scratch	-	Top	RGB	118 + 38 + 33	0.698 + 0.88 + 0.87	semantic segmentation	[130]
12	2019	USA	crack500-B + GAPs + Cracktree200 + CrackForest + Amhaz Crack	feature pyramid hierarchical boosting network	Scratch	-	Top	RGB + Laser	-	0.60 + 0.22 + 0.51 + 0.68 + 0.49	semantic segmentation	[51]
13	2020	China	crackForest + Crack500	customized U-Net	Scratch	320 × 320	Top	Intensity	72 + 1896	0.955 + 0.7327	semantic segmentation	[46]
14	2020	Canada	EdmCrack600 + CrackForest	121-layer custom CNN	Transfer	256 × 256	Back	RGB	-	0.77 + 0.92	semantic segmentation	[125]
15	2020	USA	custom + CrackForest	custom CNN (encoder + decoder) crackNet-V	Scratch	512 × 256	Top	Laser 3D images	6000	0.871 + 0.891	semantic segmentation	[131]
16	2020	USA	Aigle_RN + crackForest	custom CNN (encoder +decoder)	Scratch	48 × 48	Top	RGB	142,000 + 84,000	0.923 + 0.9533	semantic segmentation	[132]
17	2021	Iran	private (Iran)	faster RCNN + SSD	Scratch	-	Top	RGB-D	2085	0.97 *	object detection	[126]
18	2021	China	Aigle_RN + cracktree200 + crack500-B	customized U-Net with dense connection and deep supervision module	Scratch	800 × 800	Top	RGB	58 + 1896 + 206	0.65 + 0.67 + 0.64	semantic segmentation	[133]
19	2021	China	crack500-B	custom CNN model	Scratch	512 × 512	Top	RGB	1896	0.827	semantic segmentation	[134]

## Data Availability

Not Applicable.

## References

[1] Roads in Ireland—Wikipedia. https://en.wikipedia.org/wiki/Roads_in_Ireland.

[2] (2002). PASER Asphalt Roads Pavement Surface Evaluation and Rating PASER Manual Asphalt Roads. http://tic.engr.wisc.edu.

[3] Peraka N.S.P., Biligiri K.P. (2020). Pavement asset management systems and technologies: A review. Autom. Constr..

[4] Sholevar N., Golroo A., Esfahani S.R. (2022). Machine learning techniques for pavement condition evaluation. Autom. Constr..

[5] Government of Ireland (2021). Local Authority Budgets 2021. https://assets.gov.ie/139273/8554c7e7-d87c-4185-8cc1-32c8bf51c5c3.pdf.

[6] Huang J., Rathod V., Sun C., Zhu M., Korattikara A., Fathi A., Fischer I., Wojna Z., Song Y., Guadarrama S. Speed/accuracy trade-offs for modern convolutional object detectors. Proceedings of the IEEE Conference on Computer Vision and Pattern Recognition, CVPR 2017.

[7] Du Z., Yuan J., Xiao F., Hettiarachchi C. (2021). Application of image technology on pavement distress detection: A review. Measurement.

[8] Cao W., Liu Q., He Z. (2020). Review of Pavement Defect Detection Methods. IEEE Access.

[9] Ragnoli A., De Blasiis M.R., Benedetto A. (2018). Di Pavement Distress Detection Methods: A Review. Infrastructures.

[10] Coenen T.B.J., Golroo A. (2017). A review on automated pavement distress detection methods. Cogent Eng..

[11] Koch C., Georgieva K., Kasireddy V., Akinci B., Fieguth P. (2015). A review on computer vision based defect detection and condition assessment of concrete and asphalt civil infrastructure. Adv. Eng. Inform..

[12] Hou Y., Li Q., Zhang C., Lu G., Ye Z., Chen Y., Wang L., Cao D. (2021). The State-of-the-Art Review on Applications of Intrusive Sensing, Image Processing Techniques, and Machine Learning Methods in Pavement Monitoring and Analysis. Engineering.

[13] Arya D., Maeda H., Ghosh S.K., Toshniwal D., Mraz A., Kashiyama T., Sekimoto Y. (2020). Transfer Learning-based Road Damage Detection for Multiple Countries. http://arxiv.org/abs/2008.13101.

[14] Rateke T., Justen K.A., Chiarella V.F., Sobieranski A.C., Comunello E., Von Wangenheim A. (2019). Passive vision region-based road detection: A literature review. ACM Comput. Surv..

[15] Cano-Ortiz S., Pascual-Muñoz P., Castro-Fresno D. (2022). Machine learning algorithms for monitoring pavement performance. Autom. Constr..

[16] Road Pavement Surface Types. https://interpro.wisc.edu/tic/?csis-search-options=site-search&s=paser&submit=Search.

[17] Miller J.S., Bellinger W.Y. Distress Identification Manual for the Long-Term Pavement performance Program. Georgetown Pike, May 2014. https://highways.dot.gov/sites/fhwa.dot.gov/files/docs/research/long-term-pavement-performance/products/1401/distress-identification-manual-13092.pdf.

[18] Mulry B., McCarthy J. (2016). A Simplified System for Assessing the Condition of Irish Regional and Local Roads. Civ. Eng. Res. Irel..

[19] Standard Practice for Roads and Parking Lots Pavement Condition Index Surveys. https://www.astm.org/d6433-09.html.

[20] Gandhi J.R., Jaliya U.K., Thakore D.G. (2019). A Review Paper on Pothole Detection Methods. Int. J. Comput. Sci. Eng..

[21] Prasad J.R., Kanuganti S., Bhanegaonkar P.N., Sarkar A.K., Arkatkar S. (2013). Development of Relationship between Roughness (IRI) and Visible Surface Distresses: A Study on PMGSY Roads. Procedia Soc. Behav. Sci..

[22] (2020). Standard Test Method for Airport Pavement Condition Index Surveys.

[23] Mccarthy J., Fitzgerald L., Mclaughlin J., Mulry B., O’brien D., Dowling K. (2014). Rural Flexible Roads Manual—Pavement Surface Condition Index.

[24] Network Condition & Geography Statistics Branch Department for Transport, Technical Note: Road Condition and Maintenance, London, November 2021. https://assets.publishing.service.gov.uk/government/uploads/system/uploads/attachment_data/file/1032372/technical-guide-to-road-conditions.pdf.

[25] Carey W.N., Irick P.E. (1960). Highway Research Board, The Pavement Serviceability-Performance Concept. http://onlinepubs.trb.org/Onlinepubs/hrbbulletin/250/250-003.pdf.

[26] Mulry B., Feighan K., McCarthy J. Development and Implementation of a Simplified System for Assessing the Condition of Irish Regional and Local Roads. Proceedings of the 9th International Conference on Managing Pavement Assets.

[27] (2018). Federal Lands Transportation Program Instructions for FY 2019-2020 Investment Strategy (Competition). http://fltp-2019-2020-investment-strategy-guidance-2018.pdf.

[28] New Zealand, T. RAMM road condition rating and roughness manual (Manual No. PFM6). https://nzta.govt.nz/.

[29] Kazuyuki K. Pavement Maintenance in Japan. https://www.road.or.jp/international/pdf/32_AM6.pdf.

[30] Japan International Cooperation Agency, Pavement Inspection Guideline. https://openjicareport.jica.go.jp/pdf/12286001_01.pdf.

[31] Eisenbach M., Stricker R., Seichter D., Amende K., Debes K., Sesselmann M., Ebersbach D., Stoeckert U., Gross H.M. How to get pavement distress detection ready for deep learning? A systematic approach. Proceedings of the 2017 International Joint Conference on Neural Networks (IJCNN).

[32] Rateke T., Justen K.A., Von Wangenheim A. (2019). Road Surface Classification with Images Captured from Low-cost Camera-Road Traversing Knowledge (RTK) Dataset. Revista De Informática Teórica E Aplicada.

[33] Laurent J., Laurent J. Pavemetrics LCMS-Laser Crack Measurement System. https://www.pavemetrics.com/applications/road-inspection/lcms2-en/.

[34] FHWA (2018). Practical Guide for Quality Management of Pavement Condition Data Collection.

[35] Pan Y., Chen X., Sun Q., Zhang X. (2021). Monitoring Asphalt Pavement Aging and Damage Conditions from Low-Altitude UAV Imagery Based on a CNN Approach. Can. J. Remote Sens..

[36] Nappo N., Mavrouli O., Nex F., van Westen C., Gambillara R., Michetti A.M. (2021). Use of UAV-based photogrammetry products for semi-automatic detection and classification of asphalt road damage in landslide-affected areas. Eng. Geol..

[37] Inzerillo L., Di Mino G., Roberts R. (2018). Image-based 3D reconstruction using traditional and UAV datasets for analysis of road pavement distress. Autom. Constr..

[38] Saad A.M., Tahar K.N. (2019). Identification of rut and pothole by using multirotor unmanned aerial vehicle (UAV). Meas. J. Int. Meas. Confed..

[39] Zhu J., Zhong J., Ma T., Huang X., Zhang W., Zhou Y. (2022). Pavement distress detection using convolutional neural networks with images captured via UAV. Autom. Constr..

[40] Wu W., Qurishee M.A., Owino J., Fomunung I., Onyango M., Atolagbe B. Coupling Deep Learning and UAV for Infrastructure Condition Assessment Automation. Proceedings of the 2018 IEEE International Smart Cities Conference (ISC2).

[41] Biçici S., Zeybek M. (2021). An approach for the automated extraction of road surface distress from a UAV-derived point cloud. Autom. Constr..

[42] Outay F., Mengash H.A., Adnan M. (2020). Applications of unmanned aerial vehicle (UAV) in road safety, traffic and highway infrastructure management: Recent advances and challenges. Transp. Res. Part A: Policy Pract..

[43] Riid A., Lõuk R., Pihlak R., Tepljakov A., Vassiljeva K. (2019). Pavement Distress Detection with Deep Learning Using the Orthoframes Acquired by a Mobile Mapping System. Appl. Sci..

[44] Stricker R., Eisenbach M., Sesselmann M., Debes K., Gross H.M. Improving Visual Road Condition Assessment by Extensive Experiments on the Extended GAPs Dataset. Proceedings of the 2019 International Joint Conference on Neural Networks (IJCNN).

[45] Liu C., Li J., Gao J., Gao Z., Chen Z. (2021). Combination of pixel-wise and region-based deep learning for pavement inspection and segmentation. Int. J. Pavement Eng..

[46] Liu J., Yang X., Lau S., Wang X., Luo S., Lee V.C.S., Ding L. (2020). Automated pavement crack detection and segmentation based on two-step convolutional neural network. Comput. Civ. Infrastruct. Eng..

[47] Huyan J., Li W., Tighe S., Xu Z., Zhai J. (2020). CrackU-net: A novel deep convolutional neural network for pixelwise pavement crack detection. Struct. Control Health Monit..

[48] Arya D., Maeda H., Ghosh S.K., Toshniwal D., Mraz A., Kashiyama T., Sekimoto Y. (2021). Deep learning-based road damage detection and classification for multiple countries. Autom. Constr..

[49] Menegazzo J., von Wangenheim A. (2021). Road surface type classification based on inertial sensors and machine learning: A comparison between classical and deep machine learning approaches for multi-contextual real-world scenarios. Computing.

[50] Ouma Y.O., Hahn M. (2017). Pothole detection on asphalt pavements from 2D-colour pothole images using fuzzy c-means clustering and morphological reconstruction. Autom. Constr..

[51] Yang F., Zhang L., Yu S., Prokhorov D., Mei X., Ling H. (2020). Feature Pyramid and Hierarchical Boosting Network for Pavement Crack Detection. IEEE Trans. Intell. Transp. Syst..

[52] Patra S., Middya A.I., Roy S. (2021). PotSpot: Participatory sensing based monitoring system for pothole detection using deep learning. Multimed. Tools Appl..

[53] Chitale P.A., Kekre K.Y., Shenai H.R., Karani R., Gala J.P. Pothole Detection and Dimension Estimation System using Deep Learning (YOLO) and Image Processing. Proceedings of the 2020 35th International Conference on Image and Vision Computing New Zealand (IVCNZ).

[54] ROMDAS System | Road Survey Vehicle, Pavement Data Collection. https://romdas.com/romdas-system.html.

[55] Automatische Detektion von Substanzschäden mit Smart Phone und Künstlicher Intelligenz (KI)—Lehmann & Partner aus Erfurt. https://www.lehmann-partner.de/automatische-detektion-von-substanzschaeden-mit-smart-phone-und-kuenstlicher-intelligenz-ki/.

[56] Video Survey | PMSUSC. https://www.pms.ie/video-survey.

[57] Road Surface Inspection System | Global | Ricoh. https://www.ricoh.com/technology/tech/104_road_surface_monitoring.

[58] Roadway by RoadBotics. https://roadway.demo.roadbotics.com/map/wPJQ8Zc82QxFHBbswpYs/?assessmentType=normal.

[59] Gonzalez R.C., Woods R.E. (2018). 4TH EDITION Digital Image Processing.

[60] Altman N.S. (1992). An introduction to kernel and nearest-neighbor nonparametric regression. Am. Stat..

[61] Cortes C., Vapnik V.N. (1995). Support-Vector Networks. Mach. Learn..

[62] Rosenblatt F. (1958). The perceptron: A probabilistic model for information storage and organization in the brain. Psychol. Rev..

[63] Jordan M., Kleinberg J., Schölkopf B. (2006). Pattern Recognition and Machine Learning.

[64] Lowe D.G. (1999). Proceedings of the International Conference on Computer Vision.

[65] Rublee E., Rabaud V., Konolige K., Bradski G. (2011). IEEE International Conference on Computer Vision (ICCV).

[66] Alcantarilla P.F., Nuevo J., Bartoli A. Fast Explicit Diffusion for Accelerated Features in Nonlinear Scale Spaces. Proceedings of the British Machine Vision Conference (BMVC).

[67] Schmidhuber J. (2015). Deep Learning in Neural Networks: An Overview. Neural Netw..

[68] Convolutional Neural Network—Wikipedia. https://en.wikipedia.org/wiki/Convolutional_neural_network#cite_note-:0-2.

[69] Vaswani A., Shazeer N., Parmar N., Uszkoreit J., Jones L., Gomez A.N., Kaiser Ł., Polosukhin I. Attention is all you need. Proceedings of the Advances in Neural Information Processing Systems.

[70] Wang S., Tang W. Pavement crack segmentation algorithm based on local optimal threshold of cracks density distribution. Proceedings of the International Conference on Intelligent Computing.

[71] Zhao H., Qin G., Wang X. Improvement of canny algorithm based on pavement edge detection. Proceedings of the 2010 3rd International Congress on Image and Signal Processing.

[72] Othman Z., Abdullah A., Kasmin F., Ahmad S.S.S. (2019). Road crack detection using adaptive multi resolution thresholding techniques. Telkomnika.

[73] Shi Y., Cui L., Qi Z., Meng F., Chen Z. (2016). Automatic road crack detection using random structured forests. IEEE Trans. Intell. Transp. Syst..

[74] Peng L., Chao W., Shuangmiao L., Baocai F. Research on crack detection method of airport runway based on twice-threshold segmentation. Proceedings of the 2015 Fifth International Conference on Instrumentation and Measurement, Computer, Communication and Control (IMCCC).

[75] Abbas I.H., Ismael M.Q. (2021). Automated Pavement Distress Detection Using Image Processing Techniques. Eng. Technol. Appl. Sci. Res..

[76] Oliveira H., Correia P.L. CrackIT—An image processing toolbox for crack detection and characterization. Proceedings of the 2014 IEEE International Conference on Image Processing (ICIP).

[77] Zhang A., Wang K.C.P., Li B., Yang E., Dai X., Peng Y., Fei Y., Liu Y., Li J.Q., Chen C. (2017). Automated Pixel-Level Pavement Crack Detection on 3D Asphalt Surfaces Using a Deep-Learning Network. Comput. Civ. Infrastruct. Eng..

[78] Fujita Y., Shimada K., Ichihara M., Hamamoto Y. A method based on machine learning using hand-crafted features for crack detection from asphalt pavement surface images. Proceedings of the Thirteenth International Conference on Quality Control by Artificial Vision 2017.

[79] Wang S., Qiu S., Wang W., Xiao D., Wang K.C.P. (2017). Cracking classification using minimum rectangular cover--based support vector machine. J. Comput. Civ. Eng..

[80] Daniel A., Preeja V. (2014). Automatic road distress detection and analysis. Int. J. Comput. Appl..

[81] Tsai Y.C., Zhao Y., Pop-Stefanov B., Chatterjee A. (2021). Automatically detect and classify asphalt pavement raveling severity using 3D technology and machine learning. Int. J. Pavement Res. Technol..

[82] Fan Z., Wu Y., Lu J., Li W. Automatic Pavement Crack Detection Based on Structured Prediction with the Convolutional Neural Network. February 2018. http://arxiv.org/abs/1802.02208.

[83] Zou Q., Cao Y., Li Q., Mao Q., Wang S. (2012). CrackTree: Automatic crack detection from pavement images. Pattern Recognit. Lett..

[84] CRACK500—Google Drive. https://drive.google.com/drive/folders/1oJ-yoOaUf2TPbUB1LznrHOas_7imd68o.

[85] CrackForest. https://github.com/cuilimeng/CrackForest-dataset.

[86] Agile-RN and Amhaz Dataset. https://www.irit.fr/~Sylvie.Chambon/Crack_Detection_Database.html.

[87] German Asphalt Pavement Distress Dataset—GAPs | Technische Universität Ilmenau. https://www.tu-ilmenau.de/en/university/departments/department-of-computer-science-and-automation/profile/institutes-and-groups/institute-of-computer-and-systems-engineering/group-for-neuroinformatics-and-cognitive-robotics/data-sets-code/german-asphalt-pavement-distress-dataset-gaps.

[88] Arya D., Maeda H., Ghosh S.K., Toshniwal D., Sekimoto Y. (2021). RDD2020: An annotated image dataset for automatic road damage detection using deep learning. Data Br..

[89] Arya D., Maeda H., Ghosh S.K., Toshniwal D. RDD2022: A multi—National image dataset for automatic Road Damage Detection, 2020, pp. 1–16. https://arxiv.org/ftp/arxiv/papers/2209/2209.08538.pdf.

[90] Mei Q., Gül M., Azim M.R. (2020). Densely connected deep neural network considering connectivity of pixels for automatic crack detection. Autom. Constr..

[91] Lenoch0d/Road-Quality-Classification. https://github.com/lenoch0d/road-quality-classification.

[92] Ma K., Hoai M., Samaras D. Large-scale continual road inspection: Visual infrastructure assessment in the wild. Proceedings of the British Machine Vision Conference 2017.

[93] Street Pavement Rating | NYC Open Data. https://data.cityofnewyork.us/Transportation/Street-Pavement-Rating/2cav-chmn/data#revert.

[94] Pavement Condition Index (PCI). https://www.arcgis.com/apps/dashboards/5d844eacab5f40598fcd0e45376d785f.

[95] Browse the State-of-the-Art in Machine Learning | Papers with Code. https://paperswithcode.com/sota.

[96] Hong D., Gao L., Yao J., Zhang B., Plaza A., Chanussot J. (2021). Graph Convolutional Networks for Hyperspectral Image Classification. IEEE Trans. Geosci. Remote Sens..

[97] Yao J., Meng D., Zhao Q., Cao W., Xu Z. (2019). Nonconvex-Sparsity and Nonlocal-Smoothness-Based Blind Hyperspectral Unmixing. IEEE Trans. Image Process..

[98] Maeda H., Kashiyama T., Sekimoto Y., Seto T., Omata H. (2021). Generative adversarial network for road damage detection. Comput. Civ. Infrastruct. Eng..

[99] Hsieh Y.A., Tsai Y. (2021). Automated asphalt pavement raveling detection and classification using convolutional neural network and macrotexture analysis. Transp. Res. Rec..

[100] Gopalakrishnan K., Khaitan S.K., Choudhary A., Agrawal A. (2017). Deep convolutional neural networks with transfer learning for computer vision-based data-driven pavement distress detection. Constr. Build. Mater..

[101] Zhang L., Yang F., Daniel Zhang Y., Zhu Y.J. Road crack detection using deep convolutional neural network. Proceedings of the 2016 IEEE international conference on image processing (ICIP).

[102] Bhatia Y., Rai R., Gupta V., Aggarwal N., Akula A. (2019). Convolutional neural networks based potholes detection using thermal imaging. J. King Saud Univ. Inf. Sci..

[103] Yusof N.A.M., Ibrahim A., Noor M.H.M., Tahir N.M., Yusof N.M., Abidin N.Z., Osman M.K. (2019). Deep convolution neural network for crack detection on asphalt pavement. J. Phys.: Conf. Ser..

[104] Llopis-Castelló D., Paredes R., Parreño-Lara M., García-Segura T., Pellicer E. (2021). Automatic Classification and Quantification of Basic Distresses on Urban Flexible Pavement through Convolutional Neural Networks. J. Transp. Eng. Part B Pavements.

[105] Chen F.-C., Jahanshahi M.R. (2017). NB-CNN: Deep learning-based crack detection using convolutional neural network and Na{\"\i}ve Bayes data fusion. IEEE Trans. Ind. Electron..

[106] Dorafshan S., Thomas R.J., Coopmans C., Maguire M. Deep learning neural networks for sUAS-assisted structural inspections: Feasibility and application. Proceedings of the 2018 International Conference on Unmanned Aircraft Systems (ICUAS).

[107] Li B., Wang K.C.P., Zhang A., Yang E., Wang G. (2018). Automatic classification of pavement crack using deep convolutional neural network. Int. J. Pavement Eng..

[108] Li S., Zhao X. (2019). Image-based concrete crack detection using convolutional neural network and exhaustive search technique. Adv. Civ. Eng..

[109] Nguyen N.T.H., Le T.H., Perry S., Nguyen T.T. Pavement crack detection using convolutional neural network. Proceedings of the Ninth International Symposium on Information and Communication Technology.

[110] Ranjbar S., Nejad F.M., Zakeri H. (2021). An image-based system for asphalt pavement bleeding inspection. Int. J. Pavement Eng..

[111] Jenkins M.D., Carr T.A., Iglesias M.I., Buggy T., Morison G. A deep convolutional neural network for semantic pixel-wise segmentation of road and pavement surface cracks. Proceedings of the 2018 26th European Signal Processing Conference (EUSIPCO).

[112] Hsieh Y.-A., Tsai Y.J. (2020). Machine Learning for Crack Detection: Review and Model Performance Comparison. J. Comput. Civ. Eng..

[113] Long J., Shelhamer E., Darrell T. Fully convolutional networks for semantic segmentation. Proceedings of the IEEE Conference on Computer Vision and Pattern Recognition.

[114] Weng W., Zhu X. (2015). U-Net: Convolutional Networks for Biomedical Image Segmentation. IEEE Access.

[115] Zou Q., Zhang Z., Li Q., Qi X., Wang Q., Wang S. (2018). Deepcrack: Learning hierarchical convolutional features for crack detection. IEEE Trans. Image Process..

[116] Liu Z., Cao Y., Wang Y., Wang W. (2019). Computer vision-based concrete crack detection using U-net fully convolutional networks. Autom. Constr..

[117] Song W., Jia G., Zhu H., Jia D., Gao L. (2020). Automated pavement crack damage detection using deep multiscale convolutional features. J. Adv. Transp..

[118] Ronneberger O., Fischer P., Brox T. (2015). U-Net: Convolutional Networks for Biomedical Image Segmentation. Medical Image Computing and Computer-Assisted Intervention—MICCAI 2015.

[119] Badrinarayanan V., Kendall A., Cipolla R. (2015). SegNet: A Deep Convolutional Encoder-Decoder Architecture for Image Segmentation. IEEE Trans. Pattern Anal. Mach. Intell..

[120] Zhao H., Shi J., Qi X., Wang X., Jia J. Pyramid Scene Parsing Network. https://github.com/hszhao/PSPNet.

[121] Chen L.-C., Papandreou G., Schroff F., Adam H. (2017). Rethinking Atrous Convolution for Semantic Image Segmentation. arXiv.

[122] Tang Y., Zhang A.A., Luo L., Wang G., Yang E. (2021). Pixel-level pavement crack segmentation with encoder-decoder network. Measurement.

[123] Nhat-Duc H., Nguyen Q.L., Tran V.D. (2018). Automatic recognition of asphalt pavement cracks using metaheuristic optimized edge detection algorithms and convolution neural network. Autom. Constr..

[124] Bang S., Park S., Kim H., Kim H. (2019). Encoder–decoder network for pixel-level road crack detection in black-box images. Comput. Civ. Infrastruct. Eng..

[125] Mei Q., Gül M. (2020). A cost effective solution for pavement crack inspection using cameras and deep neural networks. Constr. Build. Mater..

[126] Hu G.X., Hu B.L., Yang Z., Huang L., Li P. (2021). Pavement Crack Detection Method Based on Deep Learning Models. Wirel. Commun. Mob. Comput..

[127] Abdellatif M., Peel H., Cohn A.G., Fuentes R. (2021). Combining block-based and pixel-based approaches to improve crack detection and localisation. Autom. Constr..

[128] Zhang A., Wang K.C.P., Fei Y., Liu Y., Chen C., Yang G., Li J.Q., Yang E., Qiu S. (2019). Automated Pixel-Level Pavement Crack Detection on 3D Asphalt Surfaces with a Recurrent Neural Network. Comput. Civ. Infrastruct. Eng..

[129] Konig J., Jenkins M.D., Barrie P., Mannion M., Morison G. A Convolutional Neural Network for Pavement Surface Crack Segmentation Using Residual Connections and Attention Gating. Proceedings of the International Conference on Image Processing.

[130] Li H., Song D., Liu Y., Li B. (2019). Automatic Pavement Crack Detection by Multi-Scale Image Fusion. IEEE Trans. Intell. Transp. Syst..

[131] Fei Y., Wang K.C.P., Zhang A., Chen C., Li J.Q., Liu Y., Yang G., Li B. (2020). Pixel-Level Cracking Detection on 3D Asphalt Pavement Images through Deep-Learning- Based CrackNet-V. IEEE Trans. Intell. Transp. Syst..

[132] Fan Z., Li C., Chen Y., Di Mascio P., Chen X., Zhu G., Loprencipe G. (2020). Ensemble of deep convolutional neural networks for automatic pavement crack detection and measurement. Coatings.

[133] Li H., Zong J., Nie J., Wu Z., Han H. (2021). Pavement crack detection algorithm based on densely connected and deeply supervised network. IEEE Access.

[134] Wang W., Su C. (2021). Deep Learning-Based Real-Time Crack Segmentation for Pavement Images. KSCE J. Civ. Eng..

[135] Ren S., He K., Girshick R., Sun J. (2015). Faster r-cnn: Towards real-time object detection with region proposal networks. Adv. Neural Inf. Process. Syst..

[136] Liu W., Anguelov D., Erhan D., Szegedy C., Reed S., Fu C.Y., Berg A.C. (2016). SSD: Single shot multibox detector. Computer Vision—ECCV 2016.

[137] Farhadi A., Redmon J. (2018). Yolov3: An incremental improvement. Computer Vision and Pattern Recognition.

[138] Ping P., Yang X., Gao Z. A Deep Learning Approach for Street Pothole Detection. Proceedings of the 2020 IEEE Sixth International Conference on Big Data Computing Service and Applications (BigDataService).

[139] Anand S., Gupta S., Darbari V., Kohli S. Crack-pot: Autonomous Road Crack and Pothole Detection. Proceedings of the 2018 Digital Image Computing: Techniques and Applications (DICTA).

[140] Shah S., Deshmukh C. Pothole and Bump detection using Convolution Neural Networks. Proceedings of the 2019 IEEE Transportation Electrification Conference (ITEC-India).

[141] Du Y., Pan N., Xu Z., Deng F., Shen Y., Kang H. (2020). Pavement distress detection and classification based on YOLO network. Int. J. Pavement Eng..

[142] Majidifard H., Jin P., Adu-Gyamfi Y., Buttlar W.G. (2020). Pavement Image Datasets: A New Benchmark Dataset to Classify and Densify Pavement Distresses. Transp. Res. Rec..

[143] Chen H., Yao M., Gu Q. (2020). Pothole detection using location-aware convolutional neural networks. Int. J. Mach. Learn. Cybern..

[144] Lei X., Liu C., Li L., Wang G. (2020). Automated Pavement Distress Detection and Deterioration Analysis Using Street View Map. IEEE Access.

[145] Yik Y.K., Alias N.E., Yusof Y., Isaak S. (2021). A real-time pothole detection based on deep learning approach. J. Phys. Conf. Ser..

[146] Nie M., Wang K. Pavement Distress Detection Based on Transfer Learning. Proceedings of the 2018 5th International Conference on Systems and Informatics (ICSAI).

[147] Pereira V., Tamura S., Hayamizu S., Fukai H. A Deep Learning-Based Approach for Road Pothole Detection in Timor Leste. Proceedings of the 2018 IEEE International Conference on Service Operations and Logistics, and Informatics (SOLI).

[148] Song L., Wang X. (2021). Faster region convolutional neural network for automated pavement distress detection. Road Mater. Pavement Des..

[149] Gupta S., Sharma P., Sharma D., Gupta V., Sambyal N. (2020). Detection and localization of potholes in thermal images using deep neural networks. Multimed. Tools Appl..

[150] Maeda H., Sekimoto Y., Seto T., Kashiyama T., Omata H. (2018). Road Damage Detection and Classification Using Deep Neural Networks with Smartphone Images. Comput. Civ. Infrastruct. Eng..

[151] Majidifard H., Adu-Gyamfi Y., Buttlar W.G. (2020). Deep machine learning approach to develop a new asphalt pavement condition index. Constr. Build. Mater..

[152] Rateke T., von Wangenheim A. (2021). Road surface detection and differentiation considering surface damages. Auton. Robots.

[153] Shinzato P.Y., Dos Santos T.C., Rosero L.A., Ridel D.A., Massera C.M., Alencar F., Batista M.P., Hata A.Y., Osório F.S., Wolf D.F. CaRINA dataset: An emerging-country urban scenario benchmark for road detection systems. Proceedings of the 2016 IEEE 19th International Conference on Intelligent Transportation Systems (ITSC).

[154] Fritsch J., Kuhnl T., Geiger A. A new performance measure and evaluation benchmark for road detection algorithms. Proceedings of the 16th International IEEE Conference on Intelligent Transportation Systems (ITSC 2013).

[155] Qureshi W.S., Power D., Joseph M., Brian M., Kieran F., Dympna O.S. Learning pavement surface condition ratings through visual cues using a deep learning classification approach. Proceedings of the 18th International Conference on Intelligent Computer Communication and Processing (ICCP 2022).

[156] Road-Segmentation-Adas-0001—OpenVINO Toolkit. https://docs.openvino.ai/2018_R5/_docs_Transportation_segmentation_curbs_release1_caffe_desc_road_segmentation_adas_0001.html.

[157] Tan M., Le Q. (2021). V EfficientNetV2: Smaller Models and Faster Training. https://github.com/google/.

[158] Machine Learning Datasets | Papers with Code. https://paperswithcode.com/datasets.

[159] ImageNet Benchmark (Image Classification) | Papers with Code. https://paperswithcode.com/sota/image-classification-on-imagenet?tag_filter=104%2C171%2C105.

[160] The Latest in Machine Learning | Papers with Code. https://paperswithcode.com/.

[161] Andrew Ng: Unbiggen AI—IEEE Spectrum. https://spectrum.ieee.org/andrew-ng-data-centric-ai#toggle-gdpr.

[162] Raji I.D., Bender E.M., Paullada A., Denton E., Research G., Hanna A. AI and the Everything in the Whole Wide World Benchmark. AI and the Everything in the Whole Wide World Benchmark. https://trec.nist.gov.

[163] Mathavan S., Rahman M.M., Stonecliffe-Janes M., Kamal K. (2014). Pavement raveling detection and measurement from synchronized intensity and range images. Transp. Res. Rec..

[164] Ranjbar S., Moghadas Nejad F., Zakeri H. (2022). Asphalt pavement bleeding evaluation using deep learning and wavelet transform. Amirkabir J. Civ. Eng..

[165] Hassan S., O’sullivan D., Mckeever S., Power D., Mcgowan R., Feighan K. Detecting Patches on Road Pavement Images Acquired with 3D Laser Sensors using Object Detection and Deep Learning. Proceedings of the 17th International Joint Conference on Computer Vision, Imaging and Computer Graphics Theory and Applications.

